# Supplementary figures and images for: Uncovering the organization of neural circuits with Generalized Phase Locking Analysis
Source: PLoS Comput Biol. 2023 Apr 3;19(4):e1010983. doi: 10.1371/journal.pcbi.1010983 (PMC10109521; doi:10.1371/journal.pcbi.1010983)

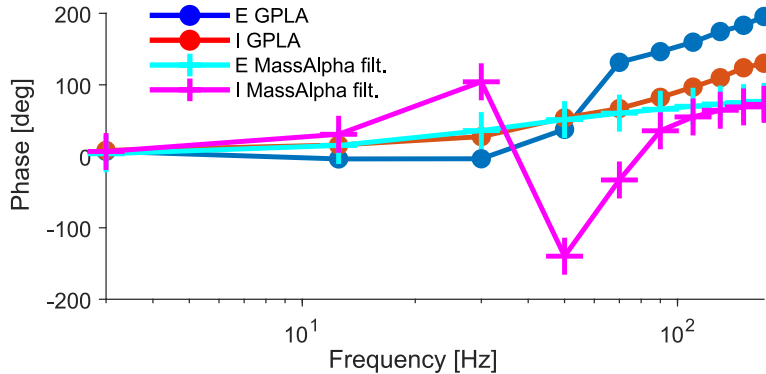

Supplement: S1 Fig — Difference between phase of excitatory and inhibitory neurons/populations based on GPLA and the excitatory and inhibitory populations in the MassAlpha neural mass model. In this simulation EPSP has been used for the LFP proxy. (PDF) [file pcbi.1010983.s002.pdf]

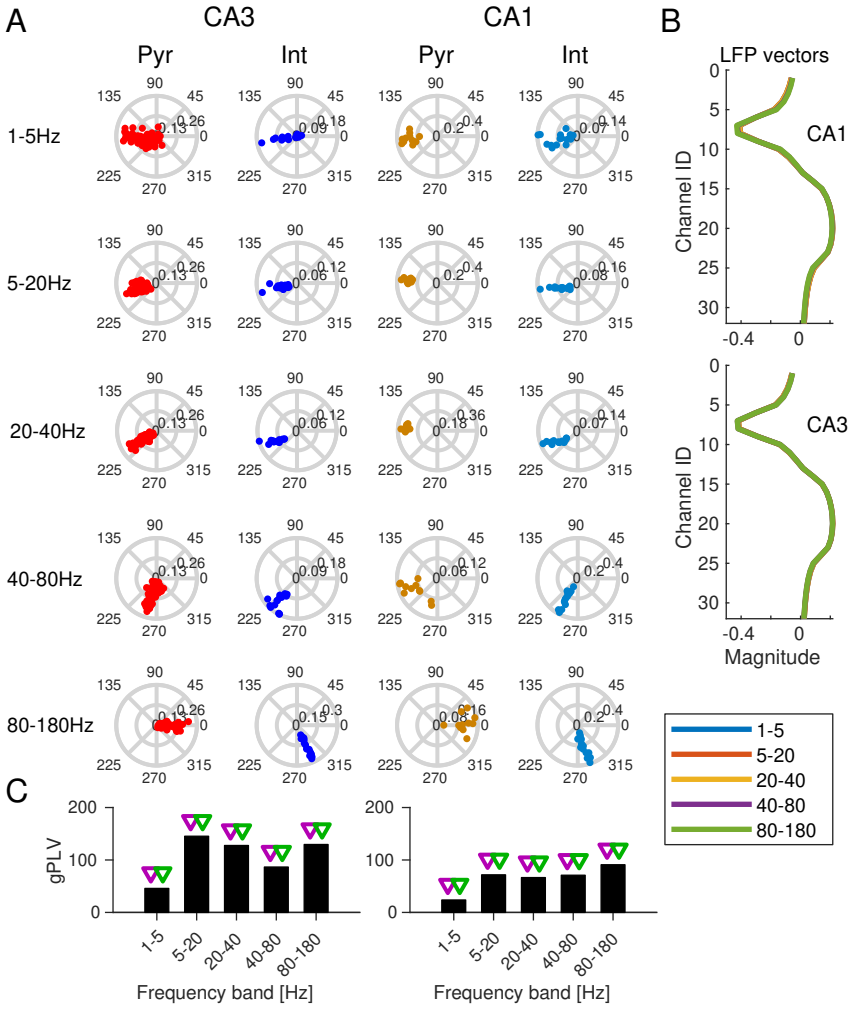

Supplement: S2 Fig — For this analysis, CA1 and CA3 data were separately injected into GPLA. (A) Spike vectors represented in polar plots similar to Fig 6E, but for all frequencies (indicated on the left). (B) LFP vectors, similar to Fig 6D, but for all frequencies (indicated in legend in the bottom). (C) gPLV for different frequency ranges of LFPs, similar to Fig 6C. Triangles indicated the significance assessed based on empirical (blue triangles, with significance threshold of 0.05) and theoretical (red triangles) tests. (left) for CA3 and (right) for CA1. (PDF) [file pcbi.1010983.s003.pdf]

**A**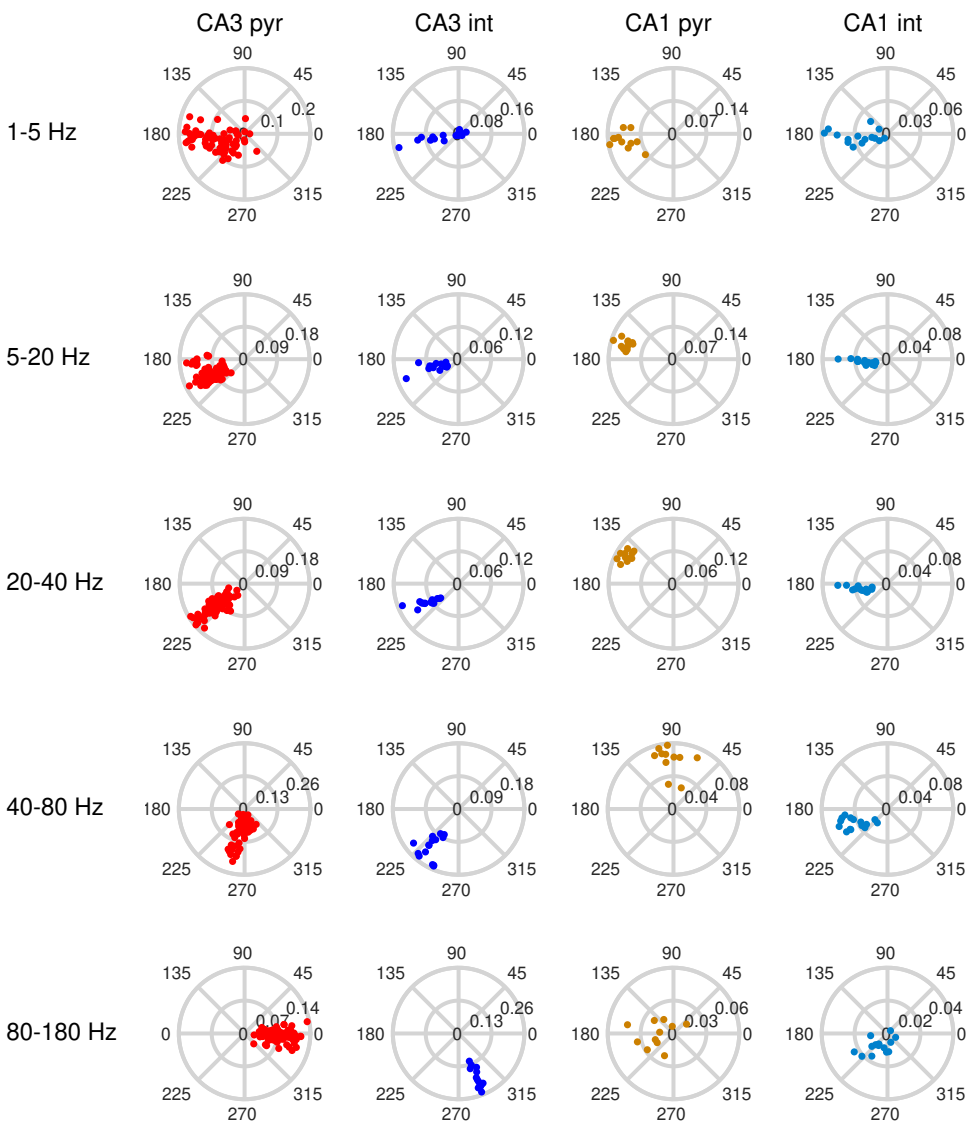

1-5 Hz

5-20 Hz

20-40 Hz

40-80 Hz

80-180 Hz

**C**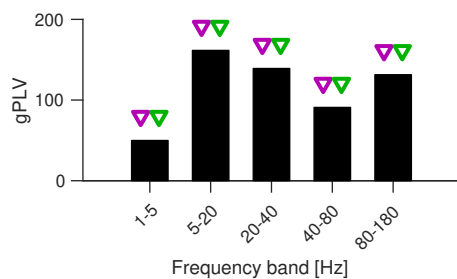**B**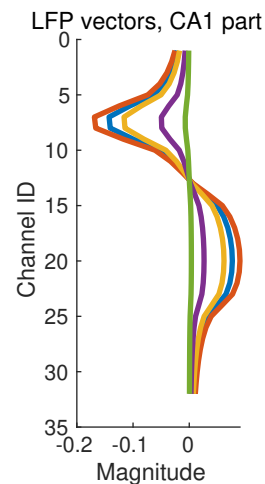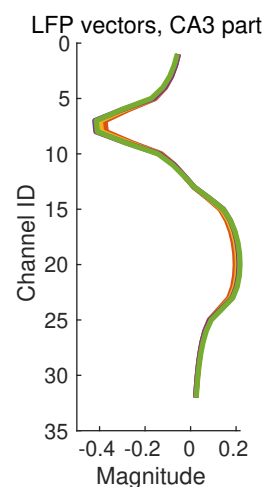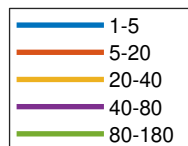

Supplement: S3 Fig — For this analysis, CA1 and CA3 data were injected to GPLA together. (A) Spike vectors represented in polar plots similar to Fig 6E, but for all frequencies (indicated on the left). (B) LFP vectors, similar to Fig 6D, but for all frequencies (indicated in legend in the bottom). (C) gPLV for different frequency ranges of LFPs Fig 6C. Triangles indicated the significance assessed based on empirical (blue triangles, with significance threshold of 0.05) and theoretical (red triangles) tests. (PDF) [file pcbi.1010983.s004.pdf]

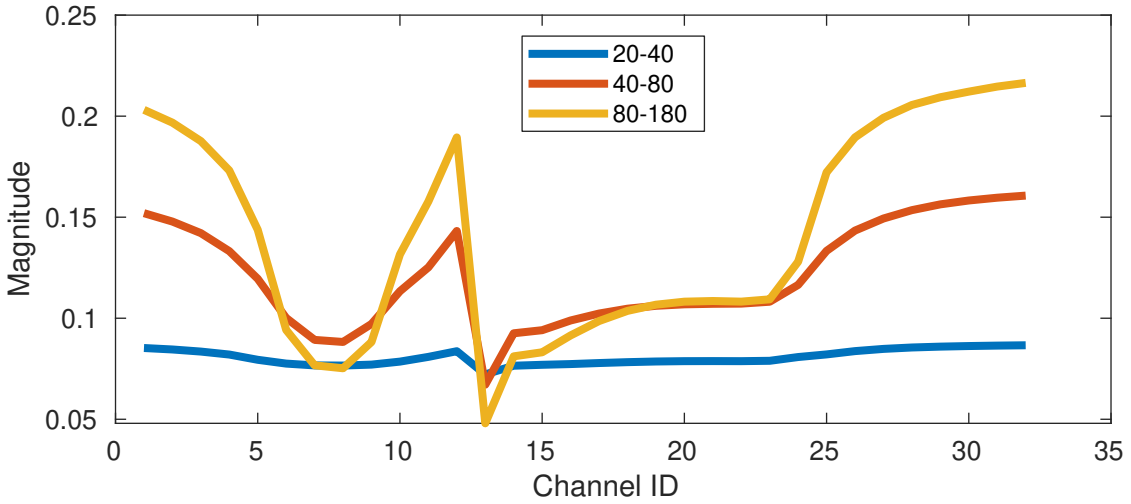

Supplement: S4 Fig — Similar to Fig 6D but based on uni-variate phase locking analysis (rather than multivariate GPLA). Each line depicts the phase locking value (PLV) for a fixed spiking units across all LFP channels. Colors indicate the frequency of filtered LFP. (PDF) [file pcbi.1010983.s005.pdf]

A

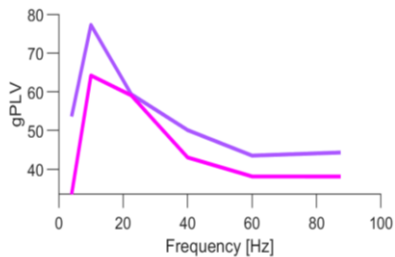

B

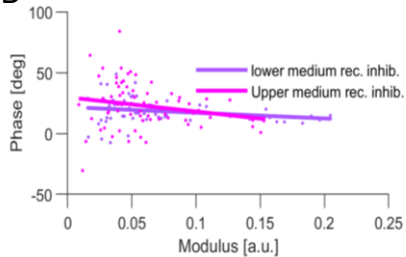

C

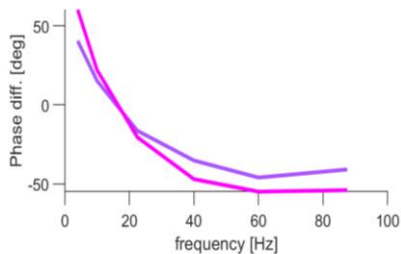

D

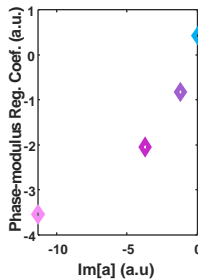

E

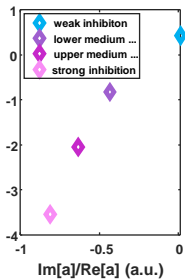

Supplement: S5 Fig — Related to Fig 7D. (A) Same as Fig 7C. for simulations at intermediate levels of recurrent inhibition. (B) Same as Fig 7D. for simulations at intermediate levels of recurrent inhibition. (C) Same as Fig 7E. for simulations at intermediate levels of recurrent inhibition. (D) Magnitude of phase modulus regression coefficient (rescaled by 180/π to have it in radians) as a function of imaginary part of a derived from Eq 9. (E) Same as (A) for Im[a]/Re[a] instead of Im[a]. (PDF) [file pcbi.1010983.s006.pdf]

**A**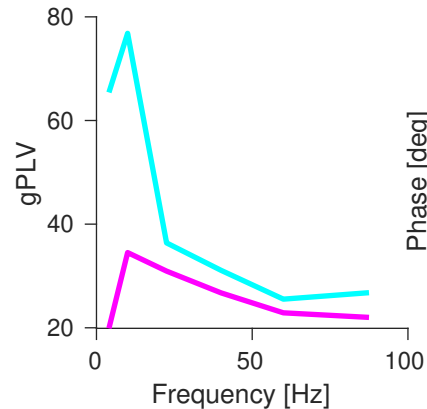**B**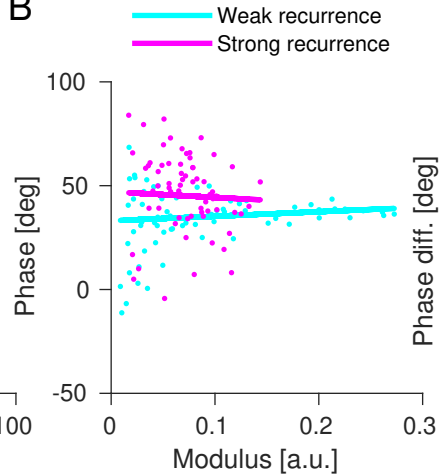**C**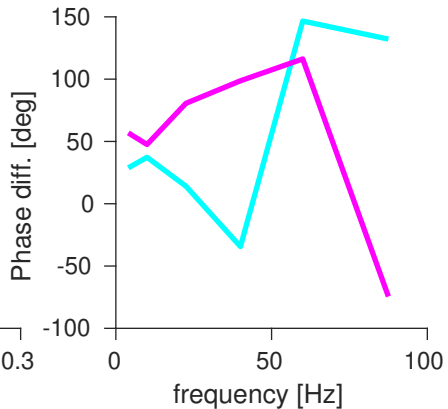

Supplement: S6 Fig — To be compared with Fig 7C–7E. (A) gPLV as a function of frequency for both models. (B) Phase of spike vector coefficients as a function of its modulus for the frequency band associated with maximum gPLV for each model (each dot one coefficient, and the continuous lines are plotted based on linear regression). (C) Shift between the averaged phase of spike vector and averaged phase of LFP vector, as a function of frequency. (PDF) [file pcbi.1010983.s007.pdf]

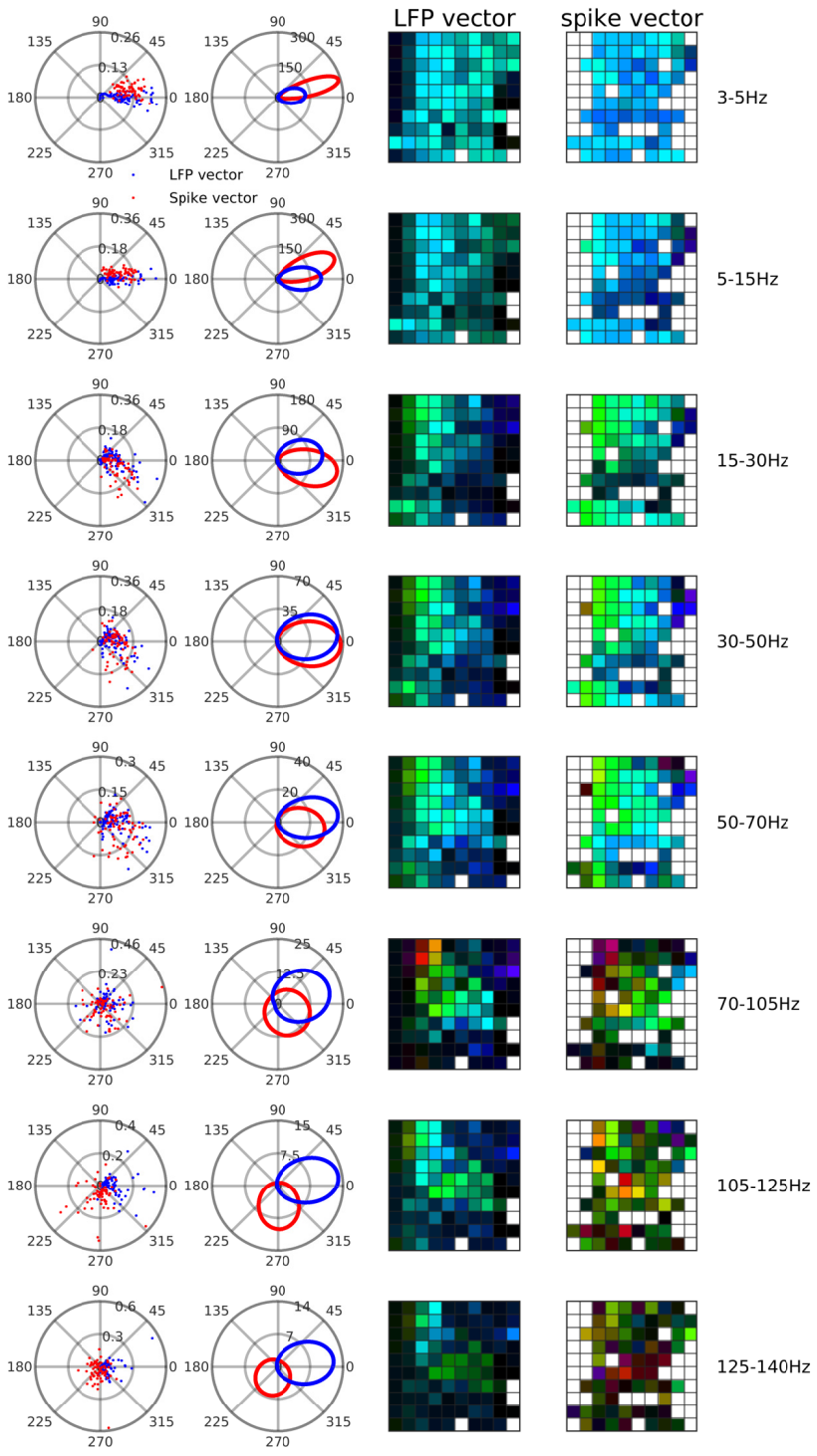

Supplement: S7 Fig — LFP and spike vectors for frequencies indicated on the right. First column depict the LFP (blue dots) and spike (red dots) in the complex plane. Second column depict fitted von Mises distribution to phase of LFP and spike vectors. Third and forth column respectively represnting phase of LFP and spike vectors which remapped to real configuration of electrodes on Utah array (see Fig 8C). (PDF) [file pcbi.1010983.s008.pdf]

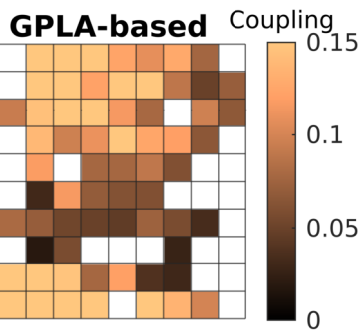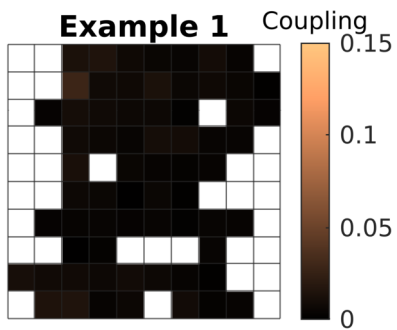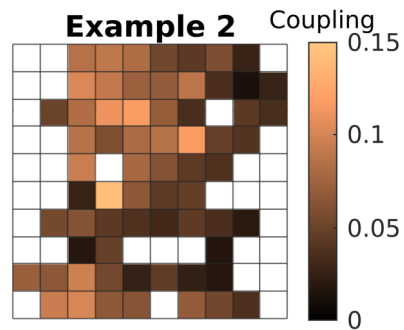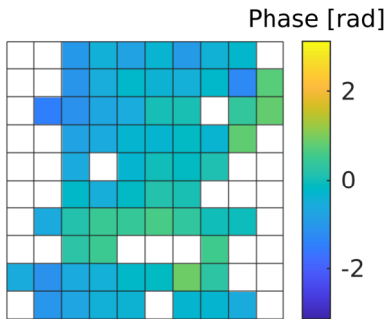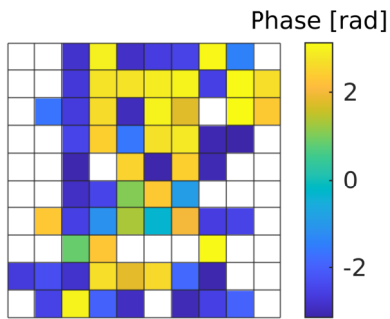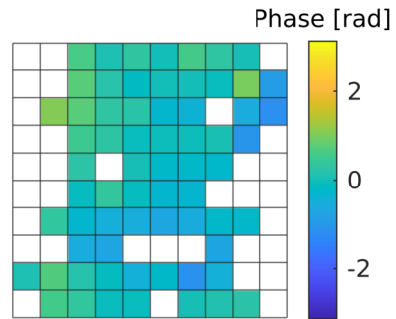

Supplement: S8 Fig — Similar to Utah array maps in Fig 8I but based on uni-variate phase locking analysis (rather than multivariate GPLA). Panels in the first row depict the spatial distribution of phase locking value (PLV) or magnitude of the spike-field coupling on the array (see Fig 8C). Panels in the second row depict the spatial distribution of locking phase on the array. White pixels in all panels indicate the recording channels with insufficient number of spikes (multiunit activity with a minimum of 5 Hz firing), as it was used in Fig 8I. The colorbars indicate the coupling strength in the first row; and locking phase in the second row. First column, depicts the results based on multivariate GPLA, and second and third column depicts the results based on uni-variate phase locking analysis, but for two different choices of LFP reference channel. The result from ‘Example 2’ is close to what is captured based on GPLA, however result from ‘Example 1’ does not, due to a lack of global coupling. (PDF) [file pcbi.1010983.s009.pdf]

**GPLA-based**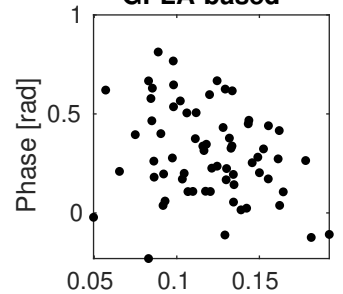**Example 1**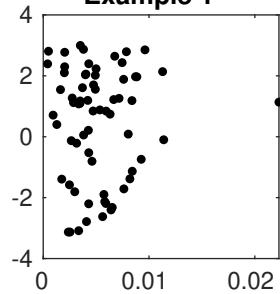**Example 2**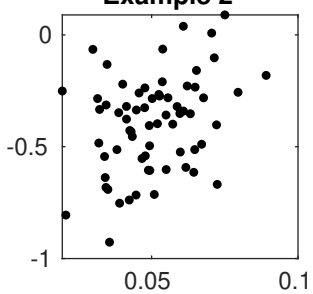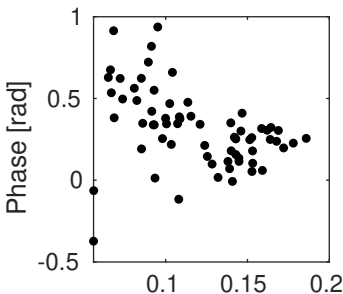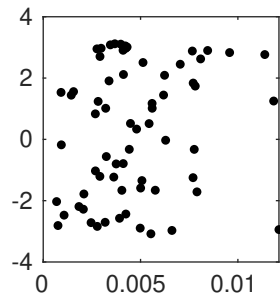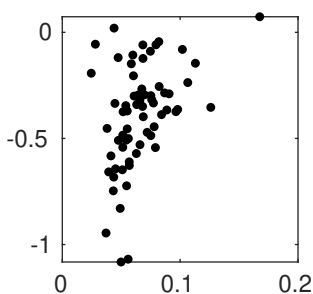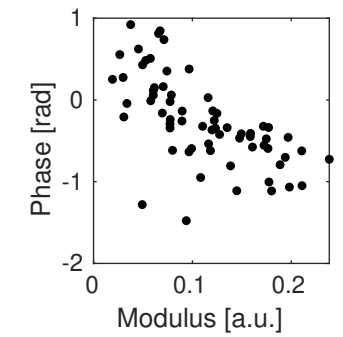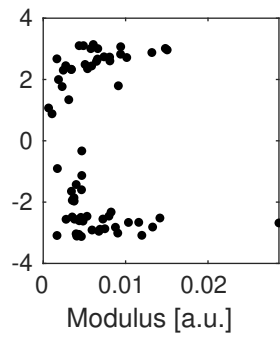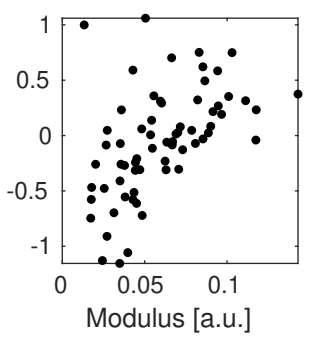

Supplement: S9 Fig — Similar to Fig 8H but based on uni-variate phase locking analysis (rather than multivariate GPLA). Each row corresponds to analysis in different frequency (the same frequencies used in Fig 8H), i. e., 3–5 Hz, 5–15 Hz, and 15–30 Hz, respectively, first, second and third row. First column indicates the results based on GPLA (notably pairwise coupling measure used here is exactly PLV), and imply the negative slope, similar to Fig 8H. The second and third columns demonstrate a similar analysis based on phase locking analysis, i. e., locking phase plotted versus strength of coupling (PLV) with two example of LFP reference channels (the same used in S8 Fig) Notably, none are compatible with our mean-field analysis (Fig 7). PLA is thus not conclusive about the strength of recurrent inhibition. (PDF) [file pcbi.1010983.s010.pdf]
