# Supplementary material for: Uncovering the organization of neural circuits with Generalized Phase Locking Analysis
Source: PLoS Comput Biol. 2023 Apr 3;19(4):e1010983. doi: 10.1371/journal.pcbi.1010983 (PMC10109521; doi:10.1371/journal.pcbi.1010983)
Supplement: Attachment — Submitted filename: gpla_plosPrep_eLifeRebuttal.pdf [file pcbi.1010983.s011.pdf]

Rebuttal of the submission to *eLife*

Dear Editors and Reviewers,

Please find below our response to the questions and comments raised by reviewers of the previous version of our manuscript, which was submitted to the journal *eLife*. The reviewers' feedback is provided in gray font, while our responses are written in blue font. To arrange for the transfer of the decision letter, and reviewer identities, the eLife editorial office (editorial@elifesciences.org) can be contacted. The title of the submitted manuscript did not change ("Uncovering the organization of neural circuits with generalized phase locking analysis") and the manuscript ID is "30-09-2021-TR-eLife-74357".

Reviewer #1
- Reviewer #1 (Public Review):
- "Reviewer #1 (Recommendations for the authors):

Reviewer #2
- Reviewer #2 (Public Review):
- Reviewer #2 (Recommendations for the authors):

Reviewer #3
- Reviewer #3 (Public Review):
- Reviewer #3 (Recommendations for the authors):

Reviewer #1

Reviewer #1 (Public Review):

This is a very interesting manuscript describing a method to compute a generalized phase-locking value (PLV), which is a multivariate extension of the standard PLV for multiple channels and neurons using singular value decomposition. gPLV estimates the main phase-locking dynamics of several neurons across LFP channels without information about their identity thus favouring an unbiased inference of neuronal dynamics. It does not only estimate the dominant spatiotemporal distribution of neuronal firing, but also the strengh of coupling. The authors present the theoretical foudation of the method and use some examples for clarifying application and methodological details. The paper is extensive and very dense at some instances, but it has merits to advance the field. I do not have any major concern with the method itself, but the ms would benefit from clarifying some issues regarding assumptions and performance of SVD. Another issue is the density and the diversity of data tested, which make difficult to convey a clear unified message. These requires substantial revision and refinement."

We have updated the manuscript in order to address the above general comments of Reviewer #1. Notably, we provide stronger links between the different sections, as described in the detailed response to the comments below.

“Reviewer #1 (Recommendations for the authors):

This is a very interesting manuscript describing a method to compute a generalized phase-locking value (PLV), which is a multivariatel extension of the standard PLV for multiple channels and neurons using singular value decomposition. gPLV estimates the main phase-locking dynamics of several neurons across LFP channels without information about their identity thus favouring an unbiased inference of neuronal dynamics. It does not only estimate the dominant spatiotemporal distribution of neuronal firing, but also the strengh of coupling. The authors present the theoretical foudation of the method and use some examples for clarifying application and methodological details. The paper is extensive and very dense at some instances, but it has merits to advance the field. This is a very good method to faccilitate analysis of high-density ephys recordings.

General comments:
- I do not have any major concern with the method itself. The fact that it is based in singular value decomposition may entail some issues regarding assumptions and performance of the algorithm. These can be simply discussed. “

> Such matters were already discussed in the discussion section, subsection “Limitations, and potential extensions”. More detailed clarifications are also provided below (in response to specific comments of the reviewer).

“For instance, there is the assumption of independency between units (their coupling will only depend on the LFP channel, and not on the rate of another neuron) which may not consider firing cross-correlation effects which are very common between pyramids-interneurons. “

> There is no assumption about the independence of firing rates in our approach. Dependence between the rates is quantified through the spiking (singular) vector. We made this point explicit in the section “Generalizing SFC to the multivariate setting” as follows:
> *“Notably, this implies that all units and all LFP channels with non-vanishing coefficients in spike and LFP vectors have correlated activities at this frequency, as will be further illustrated in the next section. In particular, two units with non-zero coefficients in the spike vector typically have correlated spike rates at this frequency.”*

Also, I assume there is no dimensionality reduction (as indicated in Fig.1) before applying SVD to the coupling matrix as this will result in value combinations. Regarding SVD operation, how computationally efficient the method is dependending on the number of channels and units? In most cases 16 or 32ch are expected, and typically one can manage tens of units. Are there some indication of how fast the SVD algorithm converges under these conditions?

> We use the standard SVD routine available in Matlab, which allows estimating the largest singular value and associated vectors. This relies on the use of Lapack routines described in their user guide (http://www.netlib.org/lapack/lug/node53.html). We use the default tolerance (1e-14) and the maximum number of iterations (300). We assessed the computational time of this elementary computation for square random matrices, which was inferior to the second for matrices of size (3000x3000), which is a much harder setting than the ones we use in our experiments (the largest case was

related to figure 7 with 225 units). We believe that for such standard algorithms benchmarking is not needed, but if the reviewers think otherwise we can of course provide it.

- One issue with the ms is its density and the diversity of data tested, which make difficult to convey a clear unified message. While it is advisable testing the method in a range of examples, some of them provide a rather reiterative perspective. The authors aim to build across levels of complexity and conceptualization, but the result if that the reader loses the focus. For instance, the toy models are useful to introduce application, but then the sections on neural field model, the generative model and spike-field phase lags (fig.5) read too theoretical. They are used later to support decisions and observations when applied to the 2D neural field model , but the way the narrative builds up is dense and memory-demanding for the reader. Similarly, the section on reduction of complex models while interesting also relies on theoretical grounds. While there is audience for these pieces, I would advocate optimizing the paper either by removing them or maybe integrating these angles into supplementary material as they are required along the narrative. This is a suggestion but the fact that the ms is dense and the number of examples is overwhelming requires careful consideration.

We do understand the concern of the reviewer and appreciate their suggestion. As we believe the readers of PLoS Computational Biology would expect the detailed analysis we have performed, we have focused on improving the writing and the coherence of the manuscript, while keeping most of the original structure. To further clarify the rationale of the simulations used, we argue that, as a methodological contribution, this manuscript needs to demonstrate the application of our approach to distinct scenarios, wherein ground-truths are available and based on which benefits can be substantiated and discussed. Therefore, we insist on keeping the wide variety of models:

(1) Toy simulations (Figure 2) illustrate the interpretability of the maps, as the method is meant to be used by experimentalists with less familiarity with multivariate approaches.

(2) Simulations of phase-locked spike trains (Figures 3 and 4) are necessary for demonstrating the statistical properties of the methods and significance assessment procedures.

(3) The study of hippocampal SWRs (Figure 6) is necessary for showcasing the performance of our approach on a topic for which spike-LFP coupling is considered a key marker of network dynamics. The use of a biophysically realistic computational model based on Hodgkin–Huxley neurons allows us to relate the mesoscopic interpretation based on simpler (neural mass) models to the biophysical ground truth.

Furthermore, we added the following paragraph in the introduction, which briefly describes the flow of the demonstration to help the reader navigate through:

“The benefits of this approach are demonstrated in detail in the results section and organized as follows. First we provide a theoretical motivation and illustrate how to interpret the outcome of the analysis with toy models. Then several analyses illustrate statistical benefits over uni-variate methods and how significance can be efficiently assessed. Thereafter, the outcome of GPLA is interpreted in term of circuit properties using an analytical reduction of two population (excitatory-inhibitory) neural field models. This mechanistic interpretation is exemplified in computational models of hippocampal and cortical neural networks. In particular, we show how studying the phase of GPLA can untangle the contribution of recurrent interactions to the observed

spatio-temporal dynamics. Based on these results, application of GPLA to Utah array recordings finally provides evidence of strong feedback inhibition in the macaque prefrontal cortex."

- One interesting toy example is using models of SWR to exemplify how gPLV may provide new insights about the spatiotemporal dynamics during a transient event (extending applicability beyond oscillations). The authors exploits two-compartment models of CA3 and CA1 neuronal populations operating with EE, EI, IE and II synaptic transmission. This model replicates some in vivo findings and in particular the firing dynamics by pyramidal cells and interneurons across different frequency bands. The analysis provides interesting insights on phase-lags between neuronal populations (which are implicitly modeled) and illustrates how the LFP vector resulting from the analysis can be interpreted in terms of current-source density signals. This is very useful.

We thank Reviewer #1 for emphasizing the interest of our modeling study of hippocampal sharp waves.

There are however some claims which should be avoided since they are based on features not included in the model or represent a use of the approach to reach conclusions on secondary issues. For instance, there is no separate afferent inputs to support claims for CA2 vs CA3. In the model the unit dynamics entirely rely in FFI. I can think of more relevant experimental features that the SWR does not include, but this is not the point though.

We assume that the reviewer meant "CA1" instead of "CA2". Separate afferent input from CA3 to CA1 was considered in the model, which does not include CA2 (that is mentioned more clearly in the revised version of result section:

"In contrast, local E-E connections are absent in CA1, but both E and I cells receive feedforward excitation from CA3".

For the joint analysis of CA1 and CA3, the afferent input remains the same as for the previous analysis of both structures taken individually. This is also stated in the revised version of the result section:

"We illustrate this by running GPLA jointly on spikes and LFPs from both CA1 and its afferent structure CA3, using the exact same model as above".

While we agree with the reviewer that additional mechanisms may be possibly investigated, however the investigated model by Ramirez-Villegas et al. (2018) is already quite detailed, and there extensive evidence in the original paper that the units dynamics does not only rely on feedforward inhibition (FFI). As stated by the reviewer, the point of the present study is not to make biological claims about hippocampal function, but to check the interpretability of GPLA when analyzing biophysically realistic activity with known ground truth mechanisms.

Another example is the use of Mass2D model which neglected FFI which is shown here to provide counter evidence of applicability for competing theories. In my opinion this should be treated in a separate piece.

We assume the reviewer refers to oscillations triggered by I-I interactions instead of FFI (as incorporating FFI did not lead to major changes). While we agree that using GPLA to evaluate competing theories deserves to be fully investigated in a separate study, we believe the qualitative results in our study provide an original perspective on the benefits of GPLA for interpreting complex high-dimensional data. We added a cautionary note in the relevant section in order to account for the reviewer's input:

“This overall suggests that GPLA combined with neural mass modeling of a structure can provide insights into the microcircuit dynamics underlying phenomena as complex as sharp-wave ripples, despite neglecting many biophysical details. While we restrict ourselves to a qualitative comparison of GPLA features for choosing which biophysical model best matches the ground truth mechanisms, this approach holds potential for designing a full-fledged GPLA-based model selection tool, whose development is left to future work.”

- The use of Utah array data as a colophon is elegant. It would be even better if it could somehow more closely relate to the previous 2D neural field models. This would make the whole ms and the different pieces a bit more cohesive.

We appreciate that Reviewer #1 noticed the interest of our analysis of Utah array data. The neural field simulations in Figure 7 are related to Utah array data in Figure 8, and it has been discussed in the last two paragraphs of the corresponding section (“Analysis of Utah array data in the prefrontal cortex”). In addition, we provide the following link at the beginning of this section:
“The interpretability of GPLA features demonstrated in the context of neural field simulations suggests that GPLA can provide mechanistic insights by analyzing spatio-temporal properties of cortical activity. Indeed, electrode arrays are able to record the activity of hundreds of units and LFP channels spatially distributed along the cortical surface, and GPLA can be used to link these activities to recurrent cortical circuits, believed to play a key role in information processing.”

Reviewer #2

Reviewer #2 (Public Review):

GPLA comments
The paper introduces a method called "generalized phase locking analysis" (GPLA) for multivariate LFP + spiking data, provides a couple of toy examples of its application on simulated data (Figs 2 +3), and develops an analytical significance criterion based on random matrix theory (fig. 4).
Then, the authors try to show how this method can be used to extract insight about circuit structures, by showing with numerical simulations how circuits with different features yield different generalized phase locking patterns (Fig. 5,6,7).
Finally they show an application to data from the ventrolateral prefrontal cortex of monkeys during visual stimulation (fig.8). They argue that GPLA shows, by comparison with the simulations in fig.7, that the circuit is a recurrent network with horizontal excitation and strong recurrent inhibition.

I think the method is interesting and has potential, however, it would be good to see more evidence of its merit when analyzing real data. The biophysical models are quite impressive (and almost "overkill" with respect to the applications) however they portray relatively simple situations in which different neurons phase lock to the LFP in relatively similar way. While the

results are interesting, it is not clear how much of these results could be obtained with standard cell-wise phase locking analysis.

We thank Reviewer #2 for the positive feedback on our method and our modeling approach. Her/his concerns are addressed in detail point by point below. Here is an overview of modeling works and their rationale:

1. Toy simulations (Figure 2) illustrate the interpretability of the maps, as the method is meant to be used by experimentalists with less familiarity with multivariate approaches.
2. Simulation of phase-locked spike trains (Figures 3 and 4) is necessary for demonstrating the statistical properties of the methods and significance assessment procedures.
3. The study of hippocampal SWRs (Figure 6) is necessary for showcasing the performance of our approach on a topic for which spike-LFP coupling is considered a key marker of network dynamics. The use of a biophysically realistic computational model based on Hodgkin–Huxley neurons allows us to relate the mesoscopic interpretation based on simpler (neural mass) models to the biophysical ground truth.

Only after this detailed justification, we believe it makes sense to provide an application on real data.

We do believe a *subset* of points we demonstrate with GPLA could also be addressed with uni-variate techniques, although we demonstrated that GPLA has multiple benefits in extracting reliable and interpretable information from multivariate data. As pointed out by Reviewer#1, GPLA “may provide new insights about the *spatiotemporal dynamics* during a transient event (extending applicability beyond oscillations).” We also emphasize that the hybrid analysis of SWR (neural mass modeling + GPLA) allows both qualitative and quantitative predictions based on crucial network parameters that are not accessible to uni-variate methods.

In more detail:

Generalizing spike-oscillation coupling analysis to the multivariate setting
The method is as follows:
- For each pair nm of LFP channel n and spike channel m, calculate the covariance between the firing rate of m and the hilbert transform of the LFP n ant frequency f. This yields the coupling matrix C(f)nm.
- Perform singular value decomposition (SVD), and retain only the first eigenvalue. The eigenvalue gives a coupling score (generalized phase locking value, GPLV), and the two eigenvectors (the spike vector and the LFP vector) can be used to extract a global relative phase of the spiking activity to the LFP.

My main problem here is with the pairwise coupling calculation. (equations 1 and 2): the author say that "This coupling measure is a modification of the Phase-Locking Value (PLV) (Ashida et al., 2010), and differs from the latter mainly through the incorporation of the amplitude of the oscillation in the averaging, and the absence of normalization by the spike rate".
I think this is the main problem of the method: it is not normalized by neither spike counts nor power of the oscillation in consideration. This could lead to high firing units and high power frequencies to dominate the SVD.

Why did the author make this choice? How would the result differ if the scores of the pairwise coupling were normalized? In my understanding this would be a fairer measure of the coupling the method tries to quantify.

The base quantity of Equation (1) is indeed not normalized in the sense that it behaves as a covariance (not a correlation). Using such covariance as the central quantity eases statistical analysis of the estimation procedure (notably in the context of generalization to multivariate data). However, the use of this quantity does not preclude applying a normalization deemed appropriate, once this quantity is estimated. In fact, we do incorporate normalization in our methodology. If our theoretical test is not used, the conventional (exactly as pointed out by the reviewer) normalization can be applied (normalization by number of spikes); If our theoretical test is intended to be used, statistical assessment relies on the use of particular kinds of normalization, described in the "method" section, subsection "GPLA for electrophysiology data". In the revised manuscript, we added Table 1, where the corresponding normalization (equations used for each figure) is noted. Furthermore, updated the main text (e.g., referring to Table 1) to clarify the procedure used and the role of normalization in section "Generalizing SFC to the multivariate setting", as follows:

"We consider the coupling defined in Equation 1 as a base quantity to explain our approach, while normalization will be addressed at this end of this section."

"In order to assess the effect size of d_1, but also to perform significance analysis, normalization of the coupling matrix C(f) is typically performed, as described in section "GPLA for electrophysiology data". In particular, the LFP time series can be whitened beforehand, the outcome of GLPA is invariant to the LFP power at each frequency. Table 1 indicates for which experiments this normalization is applied."

Related to that, the GPLV scale depends on the data, making it difficult to compare it across datasets.

Indeed, we had thought about that and had introduced an optional normalization in subsubsection "Optional normalization for gPLV" in the subsection of post-processing in the method section. In addition, normalization can be applied as mentioned in the above paragraph.

The authors stop the method at the first singular vectors, but in principle this method could be used to do a full frequency-based dimensionality reduction. This is maybe a direction for future work, but it could be useful to mention it: how much information is the method neglecting by focusing only on the first eigenmode? In which settings this is ok, and in which can be misleading?

In Safavi et al., *Neural Computation* 2021, we described a principled approach to identify the number of significant singular values, and we demonstrated an example of it in Figure 4H of the present manuscript. This can be extended further in future research.

The amount of information neglected by focusing only on the first eigenmode corresponds to the largest singular values, which highly depends on the settings under study. We have a variety of them in this manuscript. For instance, the simulation used in Figure 3 exemplifies a small loss, and for Figure 6 a large one. Of course, this loss can be quantified in different ways (e.g. the ratio of the largest singular value to the sum of all singular values). This is now further discussed in the section "Limitations and potential extensions": *"Another limitation comes from the nature of SVD, leading*

to orthogonal singular vectors that may or may not capture the properties of distinct physiological processes, which limits GPLA to capturing the dominant coupling between spikes and LFP, reflected in the largest SV and corresponding vectors. However, simulations used in Figure 4 demonstrate in simple cases, the number of significant SVs may correctly identify the number of neuronal population coupled to different rhythms. In general, the amount of information neglected by limiting the analysis to the largest singular value, highly depends on the settings under study. We have demonstrated a variety of them in this manuscript. For instance, the simulation used in Figure 3 exemplifies a small loss, and for Figure 6 a large one. Certainly, more quantitative approaches can also be taken, for instance, by quantifying this loss by the ratio of the largest singular value to the sum of all singular values."

Illustration of GPLA on toy examples
In this section the authors provide a toy example of the method with simulated data in the case of a single LFP channel and a number of neurons. In Fig 2.C and D they show that GPLA captures the coupling strength and discriminates between a population with a single phase and one with three preferred phases.
Comments:
- Fig 2B: Where are model 2 and model 4? They report only 1 and 3 as far as I can see in the rest of the figure.

This is fixed in the newer version of the manuscript.

- These are all examples with a single LFP channel, so there is no difference with the pairwise phase locking, as the authors also point out. The population coupling is just the average of the single neuron couplings, which are also all equal in this case. I think the example would better illustrate the capabilities of the method by showing toy examples with e.g. 2 LFP channels, commenting on what the single score conveys about the global phase locking.

The intention behind this simulation was to give an intuition on the output of the GPLA with examples that are *completely* understandable based on uni-variate techniques. We now clarify this in the last paragraph of the introduction as follows: *"The benefits of this approach are demonstrated in detail in the results section and organized as follows. First we provide a theoretical motivation and illustrate how to interpret the outcome of the analysis with toy models. Then several analyses illustrate statistical benefits over uni-variate methods and how significance can be efficiently assessed. Thereafter, the outcome of GPLA is interpreted in term of circuit properties using an analytical reduction of two population (excitatory-inhibitory) neural field models."*

Statistical benefits of GPLA over univariate spike-field coupling
In this section the authors repeat the simulation of the previous section, with one LFP channel and three units, and add some gaussian additive noise to the LFP. They show that the detection rate of significant phase locking coupling is higher with GPLA than with each unit taken individually, and higher than the one obtained pooling spikes together (Fig 3 A-F).
- The comparison with pooled spikes (in fig 3E) is trivial: pooling spikes with three neurons with different phase lockings should produce by definition a low global coupling, since they cancel out.

We do agree that this is an obvious case, nevertheless, it is important for practitioners to point out that there are "obvious" settings where the PLV of pooled spikes can fail

to uncover structure in the data. There are at least two reasons for having a method that does not fail in such settings. 1- If we know such cases can potentially exist, we should have methods that do not fail in already known pitfalls. 2- Even classic studies from past decades demonstrate cases where neurons fire on very different phases of network oscillation (e.g., see Klausberger et al. Nature 2003 - figure 4), therefore the setting we exemplify indeed reflects issues relevant for the analysis of experimental neural data. Furthermore, it is recommended later by the same reviewer, “What the paper would benefit the most of is a test case where the pattern of phase locking is both weak and diverse enough to defeat standard pooled or cell-wise phase locking analysis.”, in fact, this simulation represents such a case: “coupling of individual neurons leading to values at the edge of significance” (corresponding to “weak coupling”), and diverse (see Figure 3D).

- The better detection of phase locking with respect to the univariate analysis (fig 3E) is interesting, but I am not sure how the fact that a different number of spike is used (basically 3 times the spike for gPLV than for each individual unit).

Both methods use the same amount of data: data from 3 neurons each with 100 spikes. Using PLA is not conclusive about the presence of coupling, while GPLA just by providing a smart way of pooling the spikes, can reliably assess the presence of coupling.

Then, the authors use the method in a setting with 50 LFP channel and 50 neurons, varying the variance of additive Gaussian noise that is injected independently in the LFP channels.
- The authoexemplify specify how the 50 LFP channels' activities are related with one another. This is a crucial aspect of the simulation and should be reported in the main text.

Some of the method details are moved to the main text (page 22, section “Materials and Method”) in the present version of the manuscript.

- Units are all locked to the same frequency in the simulation, making the coupling matrix of rank one, so the method is able to fully recover all the signal. What would happen if this was not the case? (e.g. the coupling matrix had higher rank?)

We address the case of multiple locking frequencies in the simulations of figure 4, and the coupling matrix can be recovered by using the significant singular values. This is also mentioned in the new version of the manuscript (both in the corresponding result subsection and discussion section).

- Fig 3 H and I - The fact that H (in which the number of trial is varied) and I (in which the scale of firing rate of the units is varied) are similar demonstrates that the coupling SNR depends basically only on the total number of spikes.t Could the authors comment on that? Would this change with a normalized pairwise coupling?

This is indeed true, and in this simulation, a normalized pairwise coupling has been used (see Table 1 and the above reply to the reviewer discussing normalization issues).

- Interesting collapse of all lines together for GPLA at sigma >1. Is this something expected from the method?

We can link it to the theoretical result of our theory paper (Safavi et al., Neural Computation 2021): for large dimensional matrices, there is a threshold phenomenon

(also call phase transition in the literature) that entails that there is a sharp transition at a specific noise level: below this level, the original signal can be accurately retrieved, while above this level, the signal becomes completely indistinguishable from the noise. We now mention this interpretation in the corresponding section as follows:

"Additionally, we can observe a sharp drop of the estimation quality of the GPLA-based approach as the noise increases, likely reflecting a phase transition phenomenon in high-dimensional random matrices reported in [Safavi et al., 2021]: above some noise level threshold, singular value and vector information cannot be retrieved from noisy observations, while they can be recovered with very good accuracy above it."

Spike-field phase lags for simple network models

Here the authors illustrate two toy examples of how different circuit structures lead to different phase relationships. In Fig 5D a circuit structure without feedback that leads to a delayed response in which LFP leads, then the excitatory rate follows and then the inhibitory rate follows.

In 5E another model, with spatial recurrent excitatory inhibition and a localized exogenous current: the amplitude gradient of the input leads to a phase gradient in space.

Comments**:**

- these are all examples in which the firing rate response is linearized (see also next section). They should work well in high firing, steady-state regimes, but could fail completely in a sparse firing scenario. I think this point should be better discussed by the author.

The limitations of our assumptions are now discussed in more detail in the discussion. Furthermore, is explained in the next paragraph.

Reduction of complex models based on linear response theory

In this section the authors use linear response theory to derive linear relationships between phase and amplitude of the firing rates and the LFP, based on "transfer functions", which are basically the Green functions of the linearized problem .

Comments:

- The authors want to characterize the circuit with its transfer functions, but has two limitations:
1. It only works in a regime in which the system can be linearized (high firing, steady-state etc..)
2. If we measure the response function, we can reconstruct the linearized system, but not the non-linear original circuit.

I think these two limitations should be stated a little more clearly.

Linearization is indeed a limitation, and the reviewer's remark also points to limitations pertaining to the neural network models employed. These are now discussed in more detail in the discussion, as follows:

"Neural mass modeling has of course inherent limitations due to approximating local population activity by their mean rate, such as their typical inability to account for synchronization of spike times. However, multiple refinements of these models have been developed and offer the potential for improving biological realism. Notably, next generation neural mass models are able to capture event-related synchronization between neurons (Byrne et al., 2020) and can incorporate the dynamics of intrinsic currents that are key to modeling complex phenomena empirically observed, such as spindle oscillations (Costa et al., 2016).

In addition, neural field models can be improved in light of the knowledge about the horizontal connectivity of the structure, which may not be monotonous (for example

see recent findings on non-monotonous correlation structure in V1 (Rosenbaum et al., 2017) and PFC (Safavi et al., 2018), and heterogeneous (Qubbaj and Jirsa, 2007).

More generally, a mechanistic interpretation of GPLA results in a given structure strongly relies on the accuracy of the assumptions made to perform analytical and/or computational modeling. One aspect that entails limitations is the linear response theory on which we base our interpretations in the present work. Linearization is typically justified for a stable system exhibiting low amplitude fluctuations around its equilibrium point. However, non-linear model reduction techniques such as the Galerkin method (Marion and Temam, 1989) allow extending low dimensional, interpretable approximations of high-dimensional systems to more general settings."

- I think this technical section could be moved out of the already quite dense main text.

We do understand the concern of the reviewer, and we appreciate their suggestion. Perhaps this is a concern for the readership of eLife, nevertheless, we believe the readers of PLoS Computational Biology would expect such technical details.

Application to spike-field dynamics during sharp wave-ripples

In this section the authors simulate sharp wave ripples with an hippocampal multi compartment model, and analyze the results with GPLA.

They find that :

- There is significant coupling at each frequency band, with the strongest coupling in the ripple band Fig 6C. Since the method does not normalize for frequency power, I wonder if this is an effect of frequency power alone.

Given that the analytical signals used for GPLA are whitened, this additional normalization is not required. See Table 1 and the above answer addressing normalization.

- The preferred phase changes with frequency, and differentiates cell types (Fig 6E-H).
- They fit different neural mass models to the E-I phase difference and spike-LFP phase difference as a function of frequency (Fig 6 F and G). They conclude that to qualitatively describe the shifts in phase difference and their reversal at high frequency, a model "that introduces a lag in the post-synaptic dynamics through the use of the so-called alpha synapses", is needed.

comments:

- It would be very nice to see an application of the methods to real sharp wave ripples, producing a parallel between model and data similar to the one that they produce for utah arrays in the next to sections. Plenty of data are publicly available

We do agree it is interesting, nevertheless, we believe it should be considered for separate development beyond this manuscript. Such extensions are suitable for an independent manuscript focused on multi-variate spike-field coupling analysis of SWR, potentially yielding novel insights on hippocampal dynamics. Furthermore, it would make the present manuscript, which already contains considerable material, even longer.

- I think the author should be a bit more explicit in saying that GPLA can be used only to exclude models that do not produce the observed patterns, but cannot be used, in its present form, to do full-fledged model selection.

Thanks for pointing it out; we added a cautionary note in the relevant section in order to account for the reviewer's input:
"This overall suggests that GPLA combined with neural mass modeling of a structure can provide insights into the microcircuit dynamics underlying phenomena as complex as sharp-wave ripples, despite neglecting many biophysical details. While we restricted ourselves to a qualitative comparison of GPLA features for choosing from a restricted set of biophysical models, which best matches the ground truth mechanisms, this approach holds potential for designing a full-fledged GPLA-based model selection tool, whose development is left to future work.

- In this section, it is not clear to me what we learn on ripples from GPLA that could not have been learned (or has been) with simple phase locking analysis.

We also believe a subset of points we demonstrate with GPLA could also be achieved by uni-variate phase-locking analysis (and they were intended to be for demonstration purposes). Nevertheless, as pointed out by Reviewer#1, GPLA "may provide new insights about the spatiotemporal dynamics during a transient event (extending applicability beyond oscillations). […] The analysis provides interesting insights on phase-lags between neuronal populations (which are implicitly modeled) and illustrates how the LFP vector resulting from the analysis can be interpreted in terms of current-source density signals." In addition to what is pointed out by Reviewer #1. This section emphasizes that the hybrid analysis of SWR (neural mass modeling + GPLA) allows both qualitative and quantitative predictions based on crucial network parameters. In short, here are some links we establish between circuit properties and the outcome of GPLA:

- The laminar profile of extracellular potential generated by transmembrane synaptic currents is proportional to the LFP vector, as shown in sharp-wave ripple simulations (Figure 6D).
- I-I recurrent interactions with synaptic delays manifest themselves by phase shift reversals between the excitatory and inhibitory spike/LFP vectors as frequency increases, as shown in sharp-wave ripple simulations (Figure 6F-G).

We argue that some of these insights can be provided by uni-variate analysis but, are only obtained by guiding it with the knowledge gained from decades of electrophysiological studies of SWR (like laminar structure, key cell types in synchronization). In contrast, GPLA gives us interpretable information by processing all the recorded spikes and LFPs at once. For instance, guided by previous work on the hippocampal circuitry, one can decide to use uni-variate techniques to compute the PLV between CA1 spikes and a reference CA3 LFP channel (Buzsáki et al 2015). Nevertheless, such a priori knowledge is not required by GPLA, as it can provide the dominant coupling based *all* available pair-wise information in both structures, CA1 and CA3. We added this point in the relevant resut section as follows:
"Importantly, GPLA can also provide further insights when concurrent recordings from multiple regions are available. It allows investigating the coordination of spiking activities across structures without relying an arbitrary choice of reference LFP channel, by automatically extracting a multi-channel LFP activity (reflected by the LFP vector) that relates the most to spiking activities at a given frequency."

- SWR do emerge with diverse LFP patterns with different couplings to cell activities, see eg Reichinnek et al. 2010, Taxidis et al 2015. This could be an application where to explore beyond the first singular value, in particular if looking at real data.

We do agree it is interesting, and we do appreciate the pointers, nevertheless, we believe such extensions should be considered for separate development beyond this manuscript (we do discuss such limitations in the discussion section).

Application to spatio-temporal patterns of neural field models

In this section they look at how two different models (spatial neural fields with horizontal excitatory connections and either strong or weak local recurrent inhibition) produce different results when analyzed with GPLA. They find 4 differences:

1. A shift in the peak of GPLV to higher frequencies with strong inhibition (fig 7C)
2. A negative correlation betw. modulus and phase in strong but not weak inhibition (7D).
3. A change in sign (and a broader change) of relative phase difference with frequency in strong with respect to weak inhibition (7E).
4. A stronger spatial localization of relative phase in strong with respect to weak inhibition (7G).

I think these are nice ways to try to compare the effect of different features of the model, but to me all these differences seem more quantitative than qualitative. What would happen if inhibition strength was treated as an order parameter? Would there be a phase transition between two qualitatively distinct behaviours or a continuous change in the quantitative properties? I think this could be useful to assess the interpretability of the model.

We do agree this analysis demonstrates [clear] qualitative rather than quantitative differences, but we believe for the purpose of demonstrating the capability of the GPLA for revealing the organization of neural circuits this demonstration is sufficient. We also think treating the inhibition as an order parameter is an extremely interesting analysis for further understanding the “model” and asses the interpretability of the “model”. Nevertheless, we believe it suits more for a manuscript focused on the hybrid analysis of spatiotemporal dynamics of PFC using GPLA and neural field modeling.

Analysis of utah array data in the prefrontal cortex

I found this to be the most interesting part of the paper apart from the method itself.

Here are some comments:

- In Fig 8.G they show the GPLA values and conclude that alpha is the strongest phase locked frequency. I think this interpretation suffers from the lack of normalization of the GPLA score to both spike count and frequency power.

The normalization to spike count has been applied, and given LFPs were whitened, the normalization to frequency power was not needed.

The procedure of computing pairwise coupling is explained in the “Materials and Method” section, subsection “GPLA for electrophysiology data”, and furthermore the corresponding equations used for each figure is noted in Table 1. Furthermore, in addition to mentioned pointers, we add extra information to table 1 (like which normalization was used and if whitening was used) to ease the reader.

How does Fig 8.G compare with the power distribution in those frequencies?

Similar to one of the previous points of the reviewer, given that the analytical signals used for GPLA are whitened, this additional normalization is not required.

- They test the significance of GPLA scores for each frequency band individually against chance, with both a surrogate procedure and an analytical method. Their main claims are however mostly on the difference between frequency bands. Maybe they could comment on that, I think this could be an issue (see for example the argument about power just above).

As mentioned earlier, since the filtered signals in each frequency band were whitened, the issue raised by the reviewer regarding the difference in power across frequencies is not an issue.

- In Fig 8 (I-J-K) they show an inversion of the relative phase between spikes and LFP from low to "high" (15-30Hz) frequencies, which is interesting. They speculate that this is a hallmark of strong recurrent inhibition, because it is the same thing they see in Fig.7 for the model with strong recurrent inhibition but not for the one with weak recurrent inhibition.
I think they should be more explicit on the speculative nature of this conclusion, and in general about the fact the method gives interesting way to bridge data and models, but can give "strong" evidence only when falsifying a model, by showing it is incompatible with observation, while compatibility gives a necessary, but not sufficient condition.

We believe we conveyed the speculative nature of our claim by mentioning that, "these results ***suggest*** a neural field with excitatory horizontal connections and strong local recurrent inhibition as a plausible model for the recorded prefrontal circuits". But if the reviewer believes it still needs to be further emphasized, we will do it.

- They have a nice figure of the spatial arrangement of the preferred phases on the array (Fig. 8, I-J-K, right columns), but they do not comment much about it, just saying that the localization in the beta might be a sign of a locally active population. I think this could be analyzed more in depth in connection with the modeling sections that they have before.

We do agree that is an interesting direction to extend this analysis, nevertheless, we believe it suits more for a manuscript focused on the analysis of spatiotemporal dynamics of PFC related to its function, rather than a method paper (also given that there were various other justifications and demonstration for the method).

Reviewer #2 (Recommendations for the authors):

What the paper would benefit the most of is a test case where the pattern of phase locking is both weak and diverse enough to defeat standard pooled or cell-wise phase locking analysis.

Indeed, such scenario is already presented in the manuscript. For instance, in Figure 3A-E, we have, as mentioned in the manuscript, *“coupling of individual neurons leading to values at the edge of significance”* (corresponding to “weak coupling” as the Reviewer mentioned), and diverse (see Figure 3D); and in fact, with this simple simulation, we could demonstrate GPLA defeat standard pooled *and* cell-wise phase locking analysis. Nevertheless, if the reviewer thinks further demonstration is still needed, we can of course provide it.

Also, one puzzling aspect of the paper is, why stopping at the largest singular value? There may be very valuable information in the successive singular value/vectors, even with all the caveats mentioned in the discussion.

This is discussed in detail in response to the specific Reviewer's comments above, but briefly, In Safavi et al. Neural Computation 2021, we described a principled approach to identify the number of significant singular values (beyond the largest one), and, in the present manuscript, we demonstrate an example of it in Figure 4. Therefore, other singular values are also potentially usable in certain cases, but the methodology needs to be considerably extended in future research to fully exploit all singular values in biologically interpretable way.

Reviewer #3

Reviewer #3 (Public Review):

The authors develop the GPLA approach to quantify (1) the strength of coupling and (2) the phase relationship between a set of recorded unit spike trains and a simultaneously recorded set of local field potentials (LFP). In essence, GPLA focuses on a single frequency range of bandpassed LFPs and performs a single value decomposition of the covariance matrix between all possible pairs of spike trains - bandpassed LFPs. It then uses the first eigenvalue as an approximation to quantify the overall strength of coupling at that frequency range. It also uses the corresponding eigenvectors to quantify phase delay between spike trains and LFPs. This latter part can differentiate various groups of units with different phase delay to LFPs. The authors go to great lengths to showcase how GPLA performs in simple LFP-spikes toy models with known connectivity and in neural field models, backed by mathematical analyses wherever possible, and in a much more complex (and already published) CA3-CA1 computational model of sharp wave-ripple (SWR) LFPs. The only practical application appears at the end of the paper where they finally apply GPLA on a set of recorded LFP and units from anesthetized non-human primates presented with visual stimuli.

We have provided a fairly detailed theoretical motivation and demonstrated how the decomposition appears naturally from models of mesoscopic activity. The rationale for introducing this theoretical framework is to demonstrate the GPLA enables the biophysical interpretability of neural data. In this sense, it is a fundamental element of this work, often overlooked in the literature. As far as we are concerned with making sense of neural data, we argue that interpretability is actually a very "practical" matter.

Even though this work contains a truly impressive gamut of theoretical methodologies and analyses, it unfortunately lacks a clear and convincing demonstration of how GPLA can uncover hidden relationships between LFP and spikes. It would be crucial to show that GPLA captures relationships between LFP and spikes that a simple phase locking analysis cannot pick up (or picks up erroneously). The authors do showcase that GPLA can capture correct phase relationships and couplings in simple networks but these relationships could easily be uncovered by very simple pairwise computations. In the more complex example of the SWR model, GPLA seems to again reveal known spike-LFP couplings. And most importantly, in its only application on electrophysiological recordings, GPLA results are only described but not compared against any known biophysical relationships. So the manuscript does not present a clear argument of why it is an improvement compared with existing simpler methods.

Moreover, even the presented examples contain issues that render the accuracy of GPLA questionable. I describe those in detail directly to the authors. Moreover, the manuscript suffers from a lack of clarity. A tangential analysis of LFP-spike relationship, not related to GPLA per se, further adds in unnecessary complexity and manuscript length. As a result, I find that the presented line of analyses, though complex and extensive, are not designed to convince the reader of the advantage and usability of GPLA over existing simple pairwise (but targeted!) analyses.

The primary point is, that pairwise analyses do not suffice for data interpretation at the network level unless we have simplistic scenarios described in Figure 2 and 3. In contrast, we show that GPLA provides spike- and LFP- vectors whose spatial phase gradient reflect key microcircuit properties. Perhaps some parts of our result section did not grasp the attention of the reviewer (which are now better pointed out in the introduction to guide the reader from the beginning). We believe exactly these analyses can “convince the reader of the advantage and usability of GPLA over existing simple pairwise” methods. Specifically, our method was built based on a widely accepted knowledge about the organization of neural microcircuits (see Figure 5 and sections “Generalizing SFC to the multivariate setting” and “Neural field modeling of multivariate SFC” of the manuscript). This construction allows both qualitative and quantitative predictions based on crucial network parameters. In brief, we list several links we establish between circuit properties and the outcome of GPLA:

- the laminar profile of extracellular potential generated by transmembrane synaptic currents is proportional to the LFP vector, as shown in sharp-wave ripple simulations (Figure 6D),

- I-I recurrent interactions with synaptic delays manifest themselves by phase shift reversals between the excitatory and inhibitory spike/LFP vectors as frequency increases, as shown in sharp-wave ripple simulations (Figure 6F-G),

- feedback inhibition manifests itself by spatial phase gradients in a direction opposite to classical wave propagation, notably leading to a negative correlation between the spatial variation of phase and modulus of spike vectors, as shown in neural field simulations (Figure 7D-F),

- according to the above simulation results, the analysis of Utah array data suggests that excitatory horizontal connections and strong local feedback inhibition are plausible features of prefrontal circuits (see Figure 8H), which provided support to current theories regarding this structure (Sherfey et al., 2018).

To the best of our knowledge, this is the first methodology able to uncover such non-trivial underlying mechanisms from the multivariate spike and LFP data.

Reviewer #3 (Recommendations for the authors):

Below is a list of some major issues:

1) Firstly, it is particularly hard to follow the sequence of analyses and presented results in the paper and reach any solid conclusions. The language is rather convoluted and the reader needs to refer to a long list of appendices to understand the various steps in the process.

We addressed the raised issues by distributed changes over the manuscript text, but in particular, we implemented the following:

1. We added the following paragraph in the introduction, which briefly describes the flow of the demonstration, to help the reader navigate through: *"The benefits of this approach are demonstrated in detail in the results section and organized as follows. First we provide a theoretical motivation and illustrate how to interpret the outcome of the analysis with toy models. Then several analyses illustrate statistical benefits over uni-variate methods and how significance can be efficiently assessed. Thereafter, the outcome of GPLA is interpreted in term of circuit properties using an analytical reduction of two population (excitatory-inhibitory) neural field models. This mechanistic interpretation is exemplified in computational models of hippocampal and cortical neural networks. In particular, we show how studying the phase of GPLA can untangle the contribution of recurrent interactions to the observed spatio-temporal dynamics. Based on these results, application of GPLA to Utah array recordings finally provides evidence of strong feedback inhibition in the macaque prefrontal cortex."*
2. We moved more central method details to the main text (page 27, section "Materials and Method"), dispensing the reader to refer to the appendix.
3. To establish a better connection between the neural field simulations in Figure 7 and Utah array data in Figure 8, we add the following linking paragraph at the beginning of this section: *"The interpretability of GPLA features demonstrated in the context of neural field simulations suggests it can provide mechanistic insights about experimental recordings of spatio-temporal cortical activity. Indeed, electrode arrays are able to record the activity of hundreds of units and LFP channels spatially distributed along the cortical surface, and GPLA can be used to link these activities to recurrent cortical circuits, believed to play a key role in information processing."*

Related to that, half way through Results, the authors pivot to a detailed exploration of spike-field coupling via neural field models, simple network models and linear response theory. This takes three Results sections that seem unrelated with the rest of the paper and only add to its complexity. The focus of the manuscript should be to showcase the usability and supposed advantages of GPLA against pre-existing techniques. A detailed description of the biophysical relationships between spiking the generated LFP feels unrelated and suited for a separate publication. Particularly since, to my understanding, it reveals nothing about how GPLA performance is affected by them.

We strongly disagree with this view. As emphasized at the beginning of the paper, a major weakness of existing analysis techniques is the difficulty to interpret connectivity analysis in terms of underlying biophysical mechanisms. One could even question what an approach brings in terms of neuroscientific knowledge if it is not able to do so. As a consequence, interpretability is in our view a major aspect of the "performance" of such analysis, and in this respect deserves to be emphasized with respect to

competing approaches that do not provide such features. As a consequence, we do insist that the biophysical model aspect should be kept as a central element of this manuscript.

2) The manuscript would greatly benefit from more practical applications beyond that shown in the final figure (see more comments below). The authors could use publicly available datasets of multi-region recordings (for example from the Buzsaki lab) to try and show whether GPLA can truly uncover relationships that (1) we know are true but (2) existing methods cannot uncover correctly.

We do understand the concern of the reviewer, and we appreciate their suggestion. The readers of PLoS Computational Biology may however expect such a theoretically-flavoured paper. To further clarify the rationale of the simulations used, we should elaborate that, as a methodological contribution, we believe this manuscript needs to demonstrate the application of our approach to distinct scenarios, wherein ground truths are available and based on which benefits can be substantiated and discussed. Therefore, we insist on providing first a wide variety of the models:

(1) Toy simulations (Figure 2) illustrate the interpretability of the maps, as the method is meant to be used by experimentalists with less familiarity with multivariate approaches.

(2) Simulation of phase-locked spike trains (Figures 3 and 4) is necessary for demonstrating the statistical properties of the methods and significance assessment procedures.

(3) The study of hippocampal SWRs (Figure 6) is necessary for showcasing the performance of our approach on a topic for which spike-LFP coupling is considered a key marker of network dynamics. The use of a biophysically realistic computational model based on Hodgkin–Huxley neurons allows us to relate the mesoscopic interpretation based on simpler (neural mass) models to the biophysical ground truth.

Only after this detailed justification, we believe it makes sense to provide the application on real data. We do agree more data can improve the manuscript, nevertheless, that will make the manuscript longer which is already on the edge. We believe such extensions are interesting, but suit more for another manuscript whose focus is more on data.

3) Line 133: Please explain in the main text what is meant by "relationship" here since this is a crucial point in understanding how GPLA deals with phase delays.

We rephrased it as follows:

"Importantly, based on Equation 3, the difference between the phases of each component of ***u*** *and* ***v*** *reflects the phase lag between spiking and LFP activities for the respective channels and units."*

4) The PLV measure, against which gPLV is presented, is not explicitly defined in the text. (Nor in the citation provided). Is this the vector length from a simple phase locking analysis? Please describe it so the reader can interpret the comparisons.

PLV was mentioned at the very beginning (page 3 of the submitted version to eLife) and reference was provided for that "Phase-Locking Value (PLV) (Ashida et al., 2010) ...". Nevertheless, to help the reader we additionally provide a reference to Equation (13) in the method section.

5) Figure 3 appears to have a major issue. In the authors' definition of signal to noise ratio (SNR), the numerator is the "estimation error". This is a weird definition of SNR since it means that lower SNR in fact corresponds to better estimation! Figure 3H and 3I show that GPLA yields higher SNR than pairwise phase locking analysis. Does this mean it actually yields a higher error in estimating coupling?

Thanks for pointing it out, we clarify this all over the manuscript as *"Signal-to-Noise Ratio (SNR) is defined as the ratio of coupling strength (PLV) to estimation error (the difference between estimated PLV and the ground truth)".*

6) Paragraph starting from line 236. What is the benefit of the largest eigenvalue indicating coupling between LFPs and units? One could easily derive that just by comparing covariances or phase locking between pairs of cells-LFPs.

It is simply easier to interpret a scalar (the largest singular value), combined with two vectors that tell which unit/channels are involved and how; instead of a large matrix. Imagine you have 100 spiking units and 100 LFPs, the covariance matrix has 10000 entries, and making sense of it is highly non-trivial. In fact, we have also pointed this out in the section "Generalizing SFC to the multivariate setting": *"Although $c(f)$ is straightforward to estimate based on observed spike times, leading to the quantity noted $\hat{c}(f)$ (see Safavi et al. (2021)). However, this value provides little information about the underlying neural mechanisms, due to the largely unknown specific properties of the considered cell and recording site. Alternatively, we propose to synthesize the information provided by a large number of couplings may provide a more representative picture of the functioning of the underlying circuits. Generalized Phase Locking Analysis (GPLA) is introduced as a dimensionality reduction technique to estimate the key properties of a matrix $\mathcal{C}(f)$ consisting of the pairwise coupling between a large number of units and LFP channels."*

7) Related to the previous comment: One argument the authors make is that their multivariate method is better than pairwise analyses because it avoids multiple comparisons between all unit-LFP pairs (which due to multiple comparison p-value correction could lead to false negatives). But in most cases, one does not blindly check all possible pairs of units and LFP. For example, if one recorded, say, 20 units and 1 LFP signal from a probe in CA3 and another 20 units and 1 LFP from CA1, they wouldn't blindly compute the phase relationships between all possible 80 pairs of units-LFP. Units detected from a single electrode are usually grouped when compared to those in another brain region, or only specific unit-LFP pairs that make biophysical sense are tested. So I find the multiple comparisons argument not substantial in practice.

The reviewer's suggestion is only applicable in special circumstances (like hippocampal recording), nevertheless, it is not applicable in general settings.

8) Figure 4F. There seems to be a bifurcation-like collapse in GPLA's performance exactly at a coupling strength of 0.05. Why is that? What happens at that value that renders it such a hard threshold? The same holds for the mean squared error in 4H.

We speculate, based on the theoretical results of our other paper (Safavi et al. Neural Computation 2021), that for low coupling strength, the noise is not distinguishable from

the signal, because all the singular values fall behind the significance threshold. We believe this should not be the focus of the manuscript, but certainly interesting to be further investigated in future works.

9) I am confused by what the empirical and theoretical tests in Figures 6C and 8G show. Are the colored triangles supposed to indicate significance (as asterisks typically do)? Or do they indicate the chance baseline? If it is the former, please switch to asterisks and indicate where the chance baseline is in the bar graphs (as well as standard errors in the bars). In the latter case, is gPLV actually under the chance level and therefore NOT significant?

For the theoretical test, we have a binary significance assessment (addressing whether the coupling is significant or not) and for the empirical test, we can have the conventional p-values. To have a similar symbol for both, we use triangles (asterisks could not be used for the theoretical test). When the triangle is there, it means that gPLV in the given frequency band is significant.

10) Figures 6F-G. As the authors comment in the main text, the performance of GLPA in capturing E-I phase shifts and spike-LFP phase differences is only qualitative in nature. Not quantitative. The errors between GPLA and the neural mass models is at least 50 degrees and can go above 100 degrees! Please explain how is this showcasing GPLA's performance. It is not clear.

This error quantifies the difference between a biophysical spike model and a neural mass model, as evaluated by GPLA and linear transfer function modeling. Large differences are expected due to the strong simplification performed, and the point is that the qualitative behavior of GPLA is able to rank the plausibility of different simplified models, allowing the interpretability of computational models.

As mentioned earlier in response to Reviewer#2, we also believe a subset of points we demonstrate with GPLA could also be addressed to some extent by uni-variate phase locking analysis. Nevertheless, as pointed out by Reviewer #1, GPLA “may provide new insights about the spatiotemporal dynamics during a transient event (extending applicability beyond oscillations). […] . The analysis provides interesting insights on phase-lags between neuronal populations (which are implicitly modeled) and illustrates how the LFP vector resulting from the analysis can be interpreted in terms of current-source density signals.” In addition to what is pointed out by Reviewer #1. The hybrid analysis of SWR (neural mass modeling + GPLA) allows both qualitative and quantitative predictions based on crucial network parameters. In brief, some links we establish between circuit properties and the outcome of GPLA:

- The laminar profile of extracellular potential generated by transmembrane synaptic currents is proportional to the LFP vector, as shown in sharp-wave ripple simulations (Figure 6D).
- I-I recurrent interactions with synaptic delays manifest themselves by phase shift reversals between the excitatory and inhibitory spike/LFP vectors as frequency increases, as shown in sharp-wave ripple simulations (Figure 6F-G).

Also, it is worth mentioning that, although some insights could be gained with uni-variate techniques (and some are beyond the reach of uni-variate techniques), they rely on guiding the analysis with the knowledge gained from decades of electrophysiological studies of SWR (like laminar structure, key cell types in

synchronization etc). In contrast, GPLA gives us such insight without prior-based selection of reference channels among all the recorded spikes and LFPs.

11) Figures 6E and H. I believe the same information could be acquired by looking at individual units instead of GPLA. Again, it is unclear how its use could uncover structure that pre-existing methods could not.

The univariate analysis does not allow us to ascribe phases to individual units and thus visualize them without picking an arbitrary reference LFP, for which we are no guarantee it will faithfully reflect the dominant coherent activity with which units primarily synchronize. PLV is not a quantity associated with individual units, but only with pairs. We also update the discussion to reflect this point: *"In this study, we developed the Generalized Phase Locking Analysis (GPLA) as — to the best of our knowledge — the first biophysically interpretable multivariate method for investigating the coupling between spatially distributed spiking and LFP activity. GPLA summarizes the coupling between multiple LFP spatio-temporal patterns and multiple spiking units in a compact way. At a given frequency, the spike and LFP vectors represent the dominant LFP and spiking spatio-temporal distribution and the generalized Phase Locking Value (gPLV), characterizes the strength of the coupling between LFP and spike patterns. Some of the conclusion we draw based on GPLA are also achievable with univariate techniques, but in contrast to GPLA, they are only obtainable by guiding univariate methods with considerable amount of prior knowledge on the structure under study. For instance, univariate techniques can be used for analysis we described in Figure 6E and H, nevertheless that requires finding a suitable LFP reference channel, and finding that requires prior knowledge on Hippocampus circuitry and CA1-CA3 connectivity. An arbitrary choice of reference channel will not faithfully reflect the dominant coherent activity with which units primarily synchronize. Certainly, such caveats are even more pronounced when investigating structures with less prior knowledge and with recording techniques yielding a larger number of channels."*

12) Page 17. Last two full paragraphs describe GPLA results on two neural field models. But it is not clear whether these findings reflect ground truth.

The purpose of these simulations were to demonstrate changes in the parameters of neural field simulation that lead to changes in spatiotemporal patterns of LFP and spikes that GPLA can capture them. Theoretical analysis that is provided in the next pages, explain how these changes stem from the ground truth circuit properties. Nevertheless, it should be noted that, due to the complexity of the models, these interpretations rely on approximations (see section "Analysis and simulations of neural field models" in the Appendix).

13) The final results section is the only section where GPLA could have been used to yield a clear and convincing case for its practical application on electrophysiology data. However, once again the analyses results are just described in the text. They are not compared against any ground truth. Do the couplings and phase relationships correspond to known ones or some biophysical mechanism? What do we learn from GPLA that couldn't find out by looking at pairs of LFP-units? How does the visual stimulation presented to the monkeys, versus baseline activity, alter results?

We have shown that the qualitative results are in line with the latest result on PFC connectivity. Cortical regions, and in particular PFC, remain highly unknown structures in many respects, in particular, due to their complex, mulit-layered, microcircuit structure and highly recurrent connectivity. Our contribution aims at proposing a method to uncover some of the properties of these structures based on multivariate analysis, going beyond the clear limitation of univariate analysis in such contexts.

Minor comments:

Line 156: "Model 3" should read "Model 2". Relatedly, Figure 2B shows 4 models but only 2 models are described in the Results. Please fix.

Thanks for pointing it out. This is fixed in the newer version of the manuscript.

Figure 2B. "gPLVs for different models demonstrated in C-F" should read "C-D".

Thanks for pointing it out. This is fixed in the newer version of the manuscript.

Figure 3H and elsewhere. The standard error or standard deviation of the presented means is never shown in figures. Please add those.

They can be added, but we believe they will not add much useful information for the message of these panels, but rather create clutter which leads to difficulties in understanding the figure (in these panels there are 10 lines and 2 legends - 20 additional lines/shading and extra legends need to be added for the error bars). The point of the panel is GPLA-based estimation remains superior in a wide range of SNR. Having error bars is needed for statistical analysis of this superiority that is already addressed in panel E. Nevertheless, if the reviewer still believes that necessary we will add it.
