# Supplementary material for: Uncovering the organization of neural circuits with Generalized Phase Locking Analysis
Source: PLoS Comput Biol. 2023 Apr 3;19(4):e1010983. doi: 10.1371/journal.pcbi.1010983 (PMC10109521; doi:10.1371/journal.pcbi.1010983)
Supplement: Attachment — Submitted filename: plos_rebuttal.pdf [file pcbi.1010983.s012.pdf]

Response to the reviewers

Summary

We are grateful to the reviewer for his careful reading and precious feedback. We believe many of the concerns raised have been clarified in the revised version. We overall agree with the reviewer that biological interpretation is relative to our neural field model assumptions, and did not intend to make any claim at identifying with certainty ground truth processes associated with experimental data. Indeed, how to fill the gap between scientific models and reality remains a largely open question even after a century of epistemological debates. However, relying on model assumptions that are idealizations/simplifications of reality plays a key role in scientific explanations based on mechanistic models, and we see our work as one tool contributing to this aim. Given our extensive use of models of varying complexity, we do agree that clarifying their roles in our approach is important. As a consequence, in this revised version we introduce the notion of *biophysical interpretability*, which quantifies the ability to relate a Quantity of Interest (QoI), derived from observations, to biophysical properties of a family of biophysical models. As explained below, we have written clarifications on this point in the introduction, results, and discussion of this manuscript.

Moreover, we made sure to clarify some misunderstandings regarding the comparison of our approach to the state of the art. There are two comparisons made, which are on a different level:

- Comparisons between univariate PLV and GPLA shows that the complexity of multivariate data can be leveraged to gain statistical power, while reducing the number of QoIs to analyze. We did not intend to claim absolute superiority of GPLA relative to univariate PLV regarding biophysical interpretability, as univariate quantities are constitutive of multivariate ones and rely on the same biophysical models. This has been clarified in the results and discussion.

- In contrast, we do claim superiority with respect to few spike-field multivariate approaches in terms of biophysical interpretability, in the sense that it is to the best of our knowledge the first multivariate phase locking analysis approach that explicitly establishes connections with properties of a generic family of biophysical models. We are looking forward to see more work investigating the biophysical interpretability of multivariate techniques.

Finally, regarding the purpose of our results based on biophysical simulations: they are to be seen as qualitative elements supporting that biophysical interpretability, although relying on idealized models, is a useful feature to investigate the mechanisms underlying electrophysiological measurements. We believe this fits well with the broader aim of the paper: to attract the attention of the community on desirable properties of data analysis techniques that foster mechanistic interpretations of complex data. How to leverage this property to perform full-fledged model selection or inference is an exciting avenue for future work.

Please find below our point-by-point response to the reviewer's comments, which we identify with an italic colored font.

Reviewer 1

Reviewer Point P 1.1 — *Summary: In this paper, Safavi and colleagues present several contributions:*

1. they proposal and implement a novel algorithm to measure multivariate spike-field coupling, termed gPLA, which is essentially taking the SVD of a complex-valued matrix consisting of each univariate pairwise (neuron x LFP channel) PLV estimate at a chosen frequency. The first (largest) singular value degree is taken as the total phase locking in the measured population / LFP channels, and the respective spike and LFP singular vectors denote the relative coupling degree and phase offset of that neuron / LFP channel. 2. they test the method on a simple simulation and demonstrate that gPLA is able to detect scenarios with homogeneous / clustered / graded variation in coupling to a 1D LFP signal, and that the method is robust to noise and outperforms standard univariate measures 3. they construct two statistical tests, one based on random matrix theory and the other on surrogate (jittered spiketime) analysis, to determine whether any detected coupling is statistically significant 4. they show analytically, in a simplified and linearized model of E-I network with external input, that the coupling function in the model reduces to a multiplicative scaling of the input, and that these coupling coefficients sampled at discrete spatial locations correspond to the spike and LFP singular vectors 5. they apply the method to a spiking neural network model of hippocampal SWR with HH neurons, as well as a more complex (less assumptions than in 4) neural field model, and show that gPLA outputs correspond to certain model parameters, and / or can be used for model comparison, and are consistent with known / implicated anatomical knowledge such as hippocampal cell-type specific phase locking or prefrontal connectivity motif 6. they apply the method to Utah array recordings from NHP PFC and find heterogeneous coupling frequency and phase across the recording sites, and that gPLA output qualitatively resembles that of the neural field model with strongly recurrent inhibition

From this series of observations, the authors argue that gPLA is a superior method for estimating multivariate spike-field coupling in a systematic fashion, and that since the analysis output can be used to distinguish or make inferences about model parameters from simulated data, that it can also be applied on real neural data to gain mechanistic (i.e., anatomical or physiological) insight, which has canonically been missing where SFC analyses are concerned other than broad references to communication through coherence.

Overall, this paper is packed with a lot of interesting methodological innovations, theoretical / analytical insights, and analyses. In my opinion, the authors' high-level goal of linking the family of SFC analyses to biologically meaningful quantities such as connectivity is of great interest to the systems neuroscience community, especially those concerned with theories and neural implementation of inter-areal communication. The specific gPLA method with the SVD implementation is novel as far as I know, and has the kind of insight that makes you wonder why someone else hasn't applied SVD/PCA to the multivariate coupling matrix sooner. The analytical derivation is quite interesting, and the authors arrive at a neat result, though it requires quite a strong set of assumptions (this concern is expanded in full below). Lastly, they test their method on a series of models that increase in complexity to show that gPLA output can map to certain parameters of the models, or aid in model selection (e.g., low vs. high recurrent connectivity).

Reply: We are very grateful to the reviewer for emphasizing the interest of our perspective and highlighting its potential for the community.

Reviewer Point P 1.2 — *Despite the impressive collection of modeling and analysis results, I have reservations regarding the authors main claim, e.g., "...univariate SFC measurements [...] remain blind to the role played by the overall organization of brain structures in shaping this coupling [...]", while "GPLA [...] provide rich information to help uncover how biophysical properties shape*

network activity" (abstract L9 & L14).

Reply: Before elaborating in detail on the points raised, we want to emphasize already that we agree that this claim was misleading. It was removed from the abstract in the revised version, which now goes as follows:

> *" Despite the considerable progress of in vivo neural recording techniques, inferring the biophysical mechanisms underlying large scale coordination of brain activity from neural data remains challenging. One obstacle is the difficulty to link high dimensional functional connectivity measures to mechanistic models of network activity. We address this issue by investigating spike-field coupling (SFC) measurements, which quantify the synchronization between, on the one hand, the action potentials produced by neurons, and on the other hand mesoscopic "field" signals, reflecting subthreshold activities at possibly multiple recording sites. As the number of recording sites gets large, the amount of pairwise SFC measurements becomes overwhelmingly challenging to interpret. We develop Generalized Phase Locking Analysis (GPLA) as an interpretable dimensionality reduction of this multivariate SFC. GPLA describes the dominant coupling between field activity and neural ensembles across space and frequencies. We show that GPLA features are biophysically interpretable when used in conjunction with appropriate network models, such that we can identify the influence of underlying circuit properties on these features. We demonstrate the statistical benefits and interpretability of this approach in various computational models and Utah array recordings. The results suggest that GPLA, used jointly with biophysical modeling, can help uncover the contribution of recurrent microcircuits to the spatio-temporal dynamics observed in multi-channel experimental recordings."*

We will explain our view in the next paragraphs.

Reviewer Point P 1.3 — *My main concern is that, while I think the method itself is innovative for performing SFC analysis in a naturally multivariate way, the claim that gPLA uncovers biophysical properties / mechanisms of neural circuits is not supported. As I summarized above, I believe the authors' line of reasoning is that, since gPVA output corresponds analytically to the multiplicative transfer functions in the linearized network model, and qualitatively to various aspects of the spiking and neural field models, these insights then carry forward in a general way to real data as well. I don't think this inference logically holds for the following reason: given simulated data (from a specific circuit model, spiking or neural mass), gPLA is one of many analyses one can run on the resulting spiking and LFP data. If the result of this analysis corresponds to some aspect of the generative model, it doesn't necessarily hold that the same can be said about the real data if the generative model does not capture the necessary complexity and idiosyncrasies of the neural circuit in question (e.g., hippocampus vs. PFC vs. V1).*

Reply: We absolutely agree that adequacy between the family of models chosen for interpretability and the data is an important ingredient that conditions the empirical validity of the interpretations made.

We have clarified this at the beginning of the result section in the revised manuscript as follows:

> *" This notion of interpretability clearly depends on the model employed, and its benefits for analyzing experimental data relies on the assumption that the chosen model captures well relevant properties of the ground truth mechanism, as it is the case for any attempt at understanding empirical data through mechanistic models. The validity of this assumption can*

never be fully guarantied and which aspects should be incorporated in the model to address a particular question should be assessed based on the literature. In this work, we rely on neural field models [Jirsa and Haken, 1996], which lend themselves to analytical treatment, while still accounting for key aspects of the underlying biophysical network mechanisms."

Moreover, we made sure to reiterate this cautionary note in the discussion as follows:

> *"As mentioned when introducing the concept of biophyisical interpretability, the reliability of mechanistic interpretations drawn from GPLA crucially depends on the ability of the reduced biophysical models that we use to approximate key ground truth mechanisms underlying the data. Although no absolute guaranties can be provided, we showed in two sets of simulated data that the linearized neural field approximations provided qualitative insights in line with ground truth mechanisms, which were based on more complex (notably non-linear) models."*

Here we restrict ourselves to demonstrating with simulated data that even the use of simplified models (linearized mass models) still yield GPLA properties in qualitative agreement with the biophysical properties of the ground truth models that generated the data. However, in the choice of our simplified model, we follow common practice that can also be applied to real data: we use the neurophysiology literature to identify the key mechanisms that have been identified as crucial, and study how their incorporation improves the (qualitative) match between data and predictions. As most modeling work in Neuroscience, it is not meant to settle a matter but provides guidance for further experimental validation. In particular, biophysical properties, such that the strength of inhibitory activity, can be intervened on with modern experimental tools.

Reviewer Point P 1.4 — *Equally important, it doesn't mean other methods cannot also provide the same mechanistic insight, especially when the "insight" in question is model selection between a small number of models, e.g., categorically low or high recurrent connectivity in the neural field model. While the authors acknowledge this in the discussions, e.g., referencing the fact that similar conclusions may be drawn with univariate PLV analyses, the broader point is that the inference is only possible because the space of models is limited a priori, which, I believe, is what does the heavy lifting in providing biophysical insight.*

Reply: We absolutely agree that restriction of the model space is helpful, and this follows common scientific practice. In the epistemology literature, this relates, among others, to the concept of *research programmes* [Lakatos, 1976] following a restricted set of core hypotheses combined with auxiliary ones which are updated according to evidence. In the neurophysiology context, this is also typical of the field of oscillogenesis, where few canonical mechanisms have been pinpointed and compared. Notably, two mechanisms were named after the cell types involved in generating the oscillations: Interneuron Network Gamma, or ING, and Pyramidal Interneuron Network Gamma, or PING. We are just following representative elements of this "research programme" to illustrate how our approach can be used in the context of testing mechanistic hypotheses. We provided more context for this analysis in section Application to spike-field dynamics during sharp wave-ripples of the revised manuscript, as follows:

> *" We focus on the classical question of oscillogenesis, aiming at uncovering the circuit mechanisms responsible for the emergence of fast oscillations. The way it is addressed in the literature is paradigmatic of mechanistic questions: scientists resort to experimentation and modelling to chose between a restricted number of candidate hypotheses. Two classical*

candidate mechanisms are the Interneuron Network Gamma (ING) relying on the coupling between inhibitory interneurons under tonic excitation Whittington et al. [2000], and Pyramidal Interneuron Network Gamma (PING) relying on the interaction between excitatory principal cells and inhibitory interneurons Traub et al. [1999], Börgers and Kopell [2003]. We take advantage of these biophysically realistic hippocampal simulations, for which the ING mechanism has been shown to be the generator of high frequency activity Ramirez-Villegas et al. [2018], to assess the capability to exploit biophysical interpretability to infer which of the two above hypothesis is the right one. To do that, we will derive analytically SFC's phase in linearized neural mass models of the microcircuit activity with different levels of complexity: the simplest accounting for PING, and the more complex also accounting for ING. "

We do agree that GPLA is not yet integrated in a full-fledged model comparison tool, we further emphasize it in section Application to spike-field dynamics during sharp wave-ripples of the revised version of the manuscript as follows:

> *" Lastly, we restricted ourselves to a qualitative comparison of GPLA features for choosing from a restricted set of biophysical models, which best matches the ground truth mechanisms. This approach holds potential for designing a full-fledged GPLA-based model selection tool, whose development is left to future work. "*

Reviewer Point P 1.5 — *To put it differently, for example in the introduction, the authors state that "SFC is typically restricted to a phenomenological description" (L45), but that has nothing to do with univariate SFC methods. In theory, one can go through the entire paper and replace gPLA with standard PLV and arrive at similar biophysical insights (in fact, it's up to the authors to show that this is not the case), but only because a mechanistic model is proposed, not because gPLA is inherently different.*

It's possible that I'm putting too much emphasis on this point while the authors did not intend to imply that gPLA is somehow on higher epistemological ground than PLV. If that's the case, I recommend clarifying in the writing to clearly distinguish the two different (and very valuable) contributions: the first being the multivariate SFC method, and the second being the model-driven framework of interpreting SFC-type analyses in general, and the demonstration of its utility on the various models, leading up to the NHP analysis.

Reply: As the reviewer guessed, we did not intend to imply that GPLA in itself is on a higher epistemological ground than PLV. We believe the putative superiority of GPLA of univariate PLV regarding interpretability depends on the context. Univariate PLV quantities are intermediate elements in the calculation of GPLA, and as such have overlapping properties. In particular, if the rank one approximation turns out to be exact, univariate PLV values match components of the spike and LFP vectors of GPLA (up to multiplicative constants), and as such can be considered to have similar levels interpretability. However, when the rank one reduction is an imperfect approximation of the coupling matrix (due to finite sample estimation errors, noise or idiosyncrasies of the system), then some univariate PLV values may largely differ from the GPLA outcome. In particular, this would be the case of units that are not involved in the dominant neural dynamics captured by GPLA.

Overall, we did our best to remove any general statement regarding the superiority of GPLA in terms of biophysical interpretability, and instead put emphasis on the challenged posed by the dimensionality

of pairwise coupling information. Besides the modification of the abstract, we edited the introduction as follows:

> " *Arguably two major obstacles are (1) that common SFC measures are pairwise, which makes the information conveyed about the network increasingly difficult to grasp as the number of pairs of channels increases [Zeitler et al., 2006, Ashida et al., 2010, Vinck et al., 2010, 2012, Jiang et al., 2015, Li et al., 2016, Zarei et al., 2018] and (2) the link between SFC measurements and the underlying neural circuit mechanisms is not well understood, a problem that we will call biophysical interpretability. Elaborating on the first obstacle, pairwise analyses are arguably suboptimal for modern neural recording datasets, as state-of-the-art multichannel electrophysiology systems [Dickey et al., 2009, Jun et al., 2017, Juavinett et al., 2019] allow simultaneous recording of hundreds or even thousands of sites [Pesaran et al., 2018, Jun et al., 2017, Buzsáki, 2004, Fukushima et al., 2015]. This represents an unprecedented opportunity to study the large scale collective organization binding spiking activity of individual units with mesoscopic spatio-temporal dynamics (e. g. wave patterns [Ermentrout and Pinto, 2007, Ermentrout and Kleinfeld, 2001]), but at the same time generates high dimensional matrices of pairwise connectivity measurements from which extracting interpretable information is a challenge in itself. Moreover, statistical analysis and significance assessment of parallel spike trains is also challenging (see [Grün, 2009] for a review) and requires novel, more computationally* efficient *approaches in the high dimensional setting. Elaborating on the second obstacle, this shift in data dimensionality also offers the opportunity to go beyond the phenomenological model of synchronization between these activities, to achieve a precise account of how network properties shape the detailed spatio-temporal characteristics of collective phenomena. Such account fits the general framework of* constitutive mechanistic explanation *in Science [Salmon, 1984, Craver, 2007], where the occurrence of a phenomenon is explained based on mechanisms governing components of the system under study. However, this requires simple enough links to be established between high-dimensional observations and properties of biophysical models, which remains largely unaddressed for large-scale neural networks [Stevenson and Kording, 2011, O'Leary et al., 2015, Gao and Ganguli, 2015, Williamson et al., 2019]. To reflect this issue, we will call biophysical interpretability the extent to which quantities derived from brain activity measurements can be related to properties of a given biophysical model.* "

As presented in the replies below, we provided additional analysis to support some benefits of GPLA over univariate PLV. Additionally, we also clarified in section Neural field modeling of SFC of the revised manuscript that the biophysical interpretation of univariate PLV and GPLA rely on the same neural field model.

> " *Note that, up to this point, the developed neural field models can be used to interpret univariate as well as multivariate SFC. When the number of pairs for which SFC can be computed becomes large, a difficulty of a different nature appears: how can we synthesize the interpretations that we get from all these pairs? While ad hoc approaches for selecting relevant pairs to derive interpretations from is an option, we can try instead to establish interpretability of QoIs derived from GPLA, as we found support for its relevance for describing coupling properties of the system as a whole.*"

Reviewer Point P 1.6 — *Furthermore, I believe more comparison is needed against standard*

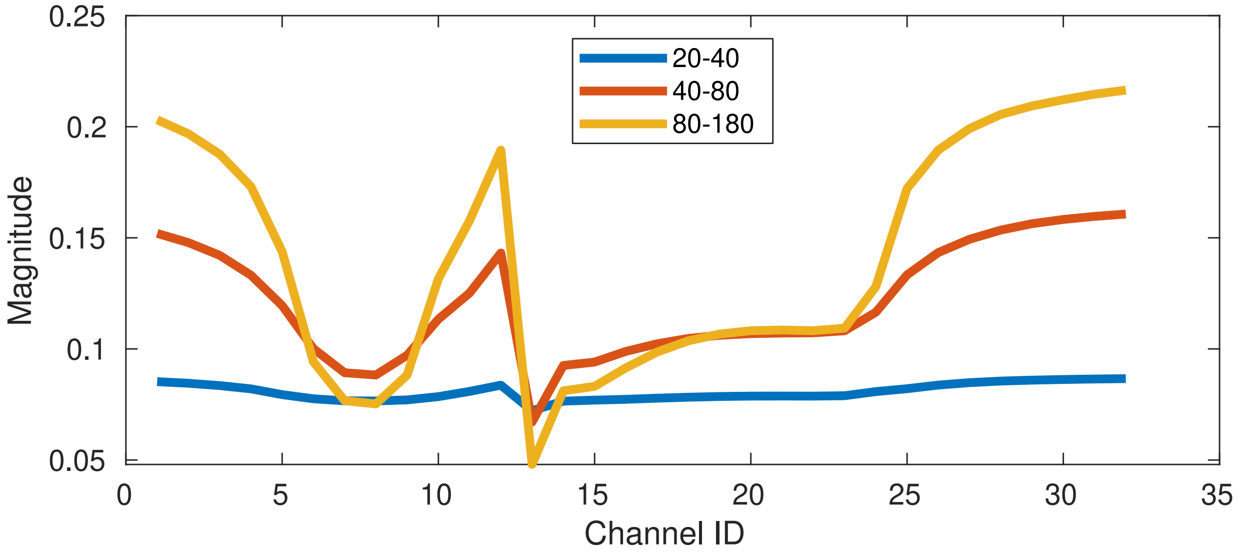

Figure 1: **GPLA vs. PLA comparison for hippocampal SWR simulation.** Similar to manuscript's Figure 6D but based on uni-variate phase locking analysis (rather than multivariate GPLA). Each line depicts the phase locking value (PLV) for a fixed spiking units across all LFP channels. Colors indicate the frequency of filtered LFP.

PLV (or some other univariate metric) for the SWR and neural field models, to show that traditional methods cannot provide the same mechanistic conclusions / differentiate between different models. Simply mentioning the fact that univariate methods require more compute and some arbitrary decisions in choosing the reference LFP channel is NOT sufficient to discount existing methods, especially if similar insights can be extracted. Such comparisons would demonstrate how gPLA (and naturally analyzing coupling in a multivariate way) is preferable to univariate methods, while I believe more emphasis should be placed in the writing that the insights are gained from the combination of gPLA AND a model that will be area- and dataset-specific, and not a generic property of gPLA.

Reply: We are grateful to the reviewer for his suggestion. We provided additional analyses and supplementary figures to address that. For the convenience of the reviewer and editor we provide the additional figures and their brief description in this rebuttal as well. In Figure 1 (labeled Supplementary 4 in the revised manuscript), we show results based on the uni-variate counterpart of GPLA (phase-locking analysis – PLA) for an fixed example unit, leads to a result that differs from the field generated by the dipolar geometric arrangement of sources and sinks in the parallel two-compartment models of pyramidal neurons used for our simulation. In contrast, the LFP vector resulting from GPLA (manuscript's Figure 6D) strongly overlap across frequency bands, and exhibit a biphasic electric potential profile typical of laminar recordings (manuscript's Figure 6D).

We also went beyond the analysis of SWR and also demonstrated the advantage of GPLA over PLA for the analysis of NHP data (as it demonstrated the benefit more clearly). In Figure 2 (labeled Supplementary Figure 8 in the revised manuscript), we demonstrate that the result of PLA is sensitive to the choice of reference channel.

In Figure 3 (labeled Supplementary Figure 9 in the revised manuscript), we further demonstrated

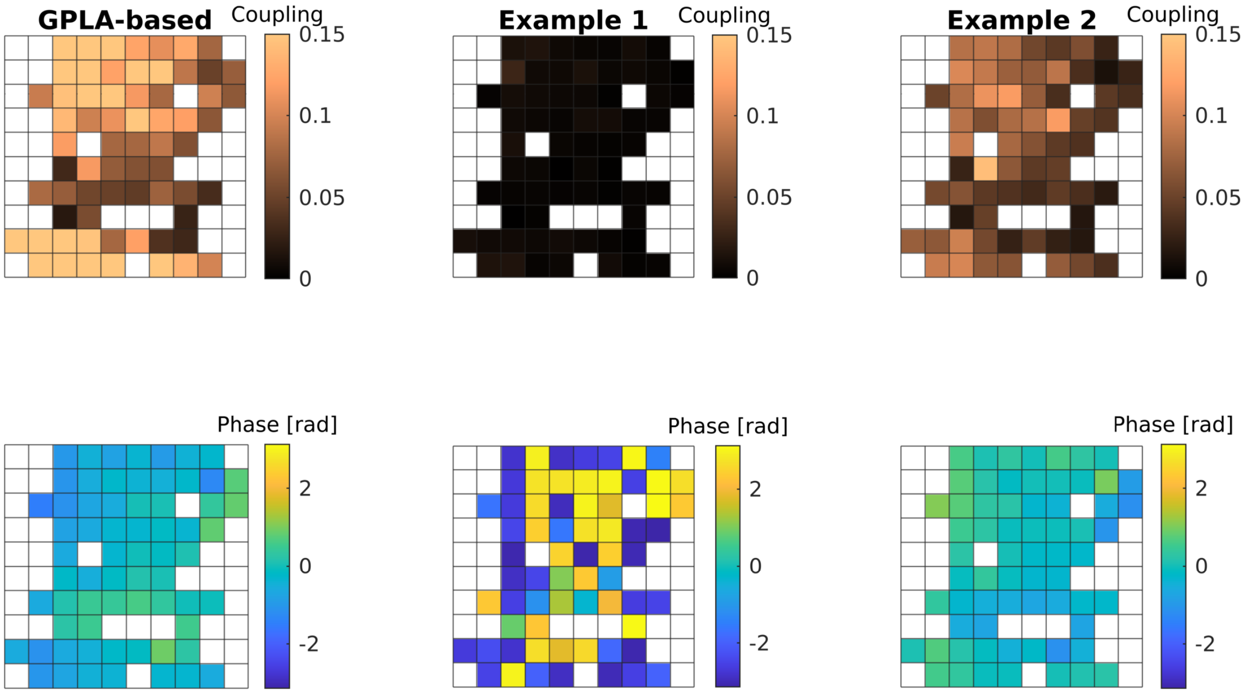

Figure 2: **GPLA vs. PLA comparison for PFC Utah array data in revealing spatial pattern of coupling.**
Similar to Utah array maps in manuscript's Figure 8I but based on uni-variate phase locking analysis (rather than multivariate GPLA). Panels in the first row depict the spatial distribution of phase locking value (PLV) or magnitude of the spike-field coupling on the array (see manuscript's Figure 8C). Panels in the second row depict the spatial distribution of locking phase on the array. White pixels in all panels indicate the recording channels with insufficient number of spikes (multiunit activity with a minimum of 5 Hz firing), as it was used in manuscript's Figure 8I . The colorbars indicate the couple strength in the first row; and locking phase in the second row. First column, depicts the results based on multivariate GPLA, and second and third column depicts the results based on uni-variate phase locking analysis, but for two different choice of LFP reference channel. The result from 'Example 2' is close to what is captured based on GPLA, however result from 'Example 1' does not, due to lack of global coupling.

other characteristic advantages of GPLA with respect to PLA. We showed the relationship between the spatial variations of modulus and phase of the spike vector is different across networks with different amounts of recurrent inhibition. In the simulation with strong recurrent inhibition, the phase of spike vector coefficients as a function of their modulus indicates that the phase of the spike vector coefficients decreases (i.e. the oscillation lags further relative to the LFP) for larger modulus, whereas, in the simulation with weak recurrent inhibition, phase is not correlated with the modulus (manuscript's Figure 7D), we demonstrate a similar behavior for NHP data (Figure 8H). In Figure 3 (Supplementary Figure 9 in the revised manuscript), we demonstrate this is only achievable through GPLA.

Reviewer Point P 1.7 — *Furthermore, if one takes this route, it would be preferable that each model is always paired with real data, which currently, only the neural field model does (loosely with the NHP data). I suppose the theoretical derivations centered on the reduced linear model does not require real data analysis (even though that would go towards justifying the assumptions made in the model), but the SWR analysis could be demonstrated on one of the many hippocampal datasets that's openly available (e.g., on CRCNS).*

Reply: We are grateful for this suggestion. For SWR analysis we need data-sets with single units, LFPs, and two classes of labels, to be able to make the correspondence with the modeling side: one category is the cell-type labels, and the other is the time-points of SWR. However, unfortunately, we could not find any of the open data-set that has all the necessary information in a single data-set. Additionally, we believe such extensions are interesting but more suitable for an independent manuscript focused on multi-variate spike-field coupling analysis of SWR (rather than introducing a methodology), potentially yielding novel insights on hippocampal dynamics. Lastly, it would make the present manuscript, which is on the edge in terms of length, even longer.

We hope that the extra analysis provided on SWR and neural field simulations as well as NHP data provide a satisfactory trade-off.

Reviewer Point P 1.8 — *On the other hand, if the authors do intend to suggest that gPLA somehow inherently allows more access to biophysical / mechanistic interpretations of the data (as is currently my reading of, e.g., L72-79, and L144-146), and is generalizable beyond the specific set of generative models they used in conjunction with the analysis method itself, then I have a number of concerns regarding the validity of such inference when strong assumptions / restrictions are made about the model:*

“

“

- I believe a central piece of evidence that allows gPLA to make mechanistic links is the fact that the spike and LFP singular vectors correspond to the transfer functions in the linearized E-I neural mass model. While the analytical derivation is beautifully done, ultimately, this is an extremely impoverished model of the cortex, as it seems to me that the full set of assumptions essentially reduces the model to a linear network without recurrent inhibitory connections. And since the LFP is computed from the current fluctuations of the exc population, the model is basically a 1-population excitatory network driven by oscillatory external input, and inhibition can be ignored entirely. I think further discussion is warranted on if and whether the theoretical conclusions drawn from this model can be extended to more complex configurations; if so, how, and if not, does this undermine the validity of the analytical results (note that this is just about the derivations and analytical

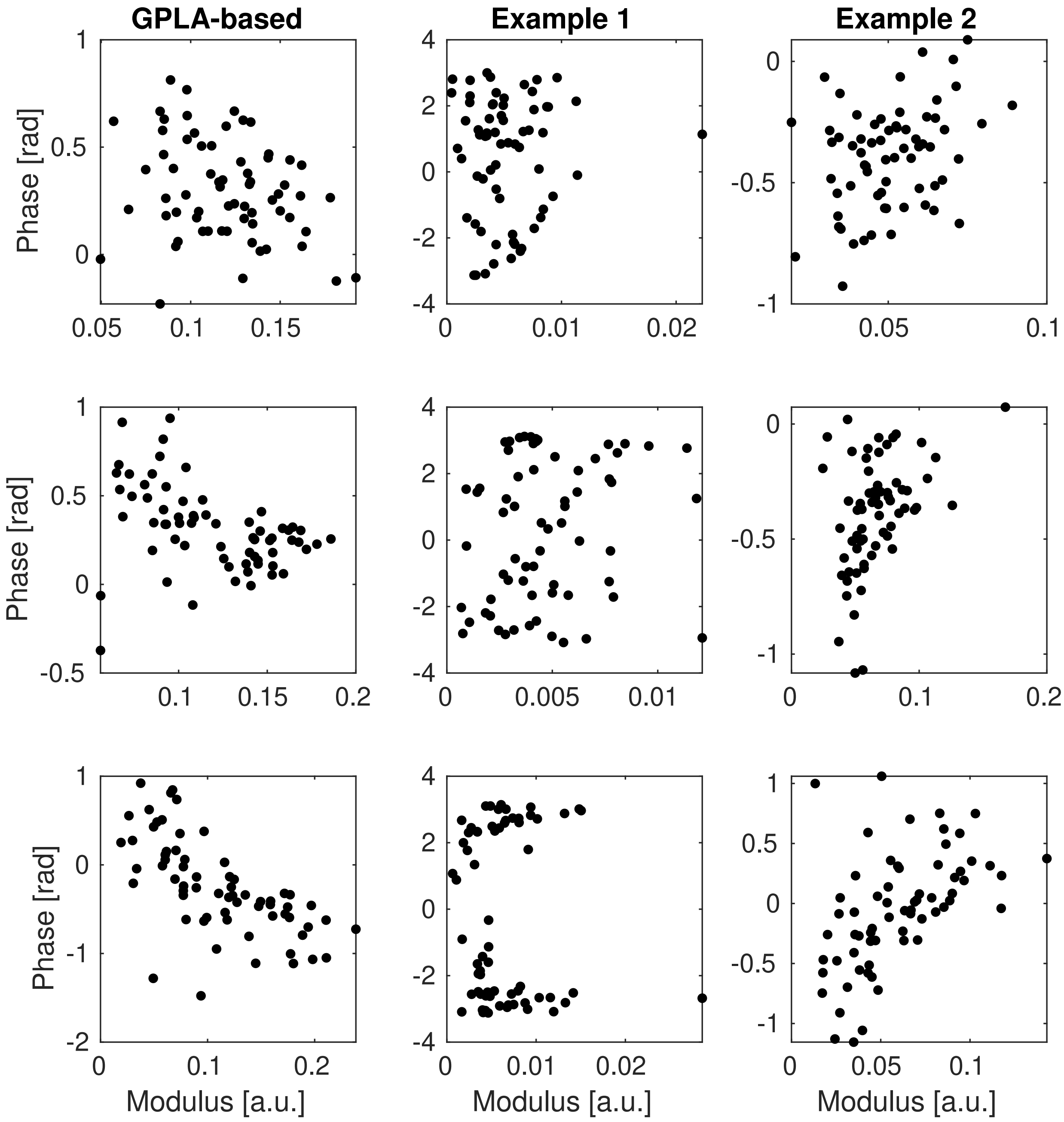

Figure 3: **GPLA vs. PLA comparison for PFC Utah array data in characterizing strength of recurrent inhibition in PFC circuit.**
Similar to manuscript's Figure 8H but based on uni-variate phase locking analysis (rather than multivariate GPLA). Each row correspond to analysis in different frequency (the same frequencies used in manuscript's Figure 8H), i. e. , 3-5 Hz, 5-15 Hz, and 15-30 Hz, respectively, first, second and third row. First column indicates the results based on GPLA (notebely pairwise coupling measure used here is exactly PLV), and imply the negative slope, similar to manuscript's Figure 8H. The second and third columns demonstrate a similar analysis based on phase locking analysis, i. e. , locking phase plotted versus strength of coupling (PLV). Two example of LFP reference channels (the same references used in manuscript's Figure supplement 8) Notably, none are compatible with our mean-field analysis (manuscript's Figure 7) , thus not conclusive about the strength of recurrent inhibition.

argument, as the later models increase in complexity in an empirical way but do not convincingly show the same analytical insight) .

Reply: Thank you for pointing out this concern. We think this likely due to a misunderstanding that we will try to clarify. The general neural field model we are considering is provided in section and does include multiple populations (one E and one I in most of our study). As far as we can tell, this model is not reducible to a one population model, as even when the contribution of inhibitory activity to the LFP is neglected, the E-I interactions do contribute to shaping the activity of the E population. In particular, such a simple two population model can generate oscillatory activity, while the E population alone cannot. In addition, please note that the contribution of the I activity to the LFP is not always assumed negligible: the manuscript's Supplementary Figure 5 compares the outcome of GPLA using a purely inhibitory LFP, and it is argued that phase analysis can be used to help identify which assumption regarding the origin of LFP is the most likely. Finally, the case of recurrent E-E and I-I interactions has a special treatment because in the case of the linear neural mass model (or the case of neural field with only local interactions), the contribution of those recurrent interactions can be absorbed in the membrane time constant. This yield a reduction of the effective number of parameters of the system, although the contribution of these interactions could still be exploited in some settings, for example by observing how changing self-excitation modifies the "apparent" membrane time constant of the E population. Of course, this can also be seen as a limitation of the model. In particular, this prevents the I-I interaction to affect qualitatively the model dynamics, and this is why we resort to the addition of an extra alpha neuron to allow this mechanism to manifest itself, and account for the ING dynamics reported in the literature. Overall, across our simulations, we go through many variants of the linearized neural field equations to demonstrate that despite its relative simplicity it can qualitatively capture features of key neural circuit mechanisms at the population level. This happens despite simulated data being generated by more complex models, i.e. Hudgkin-Huxley spiking neurons (for SWR simulations) and non-linear neural field models (for analyses of Figure 7).

Reviewer Point P 1.9 — *- for the SWR model, it appears that gPLA reproduces experimental observations of laminar current profile and cell-type specific coupling phase in Fig 6A-D. However, it's a bit unclear to me why the authors chose to add an alpha synapse / delay in the I-to-I connections, among many other possible model ingredients. By adding this component to the Mass2D model, MassAlpha qualitatively produces the biphasic shape of E-I phase shift across frequencies, but does this really imply that gPLA arrives at the "correct" mechanistic conclusion? Suppose some other biophysical detail was added to the model, such as the spatial distribution of synapses along the hippocampal layers, and that it also produces a similar shape as gPLA in Fig 6F, how would one differentiate between the competing hypotheses? While it's promising that gPLA "with the appropriate simulations capture key characteristics of the underlying circuitry" (L451, again in L466), it also does not rule out the possibility that some other (univariate) SFC metric could do the same. Again, this does not take away from gPLA's value as a multivariate analysis tool, but such a possibility would argue against the authors' claim that it uniquely reveals mechanistic insight when combined with the appropriate model.*

Reply: We hope we clarified in the reply to the above Point P 1.4 and related changes why the alpha synapse was added. We can never exclude other putative mechanism, but follow the dominant hypotheses of the literature. Please refer to the reply to the next point for further elaborations.

Reviewer Point P 1.10 — *- similarly, for the neural field model, the authors essentially restrict*

the model space to two discrete possibilities: weak vs. strong recurrent inhibition. It is promising that gPLA output can distinguish between these two possibilities, but that does not imply that gPLA output provides mechanistic insight, especially in real data. It's implied in the comparison with the real data that PFC connectivity is more consistent with the strongly recurrent model, but to what degree? At the minimum, the authors should vary inhibitory connectivity strength in a graded fashion and show that gPLA output scales parametrically, thereby providing more support for making an inference where, e.g., the slope of phase difference across frequencies corresponds to inhibitory connection density in a mechanistic way. But even then, the authors would have to explain why 1) no other model parameters would strongly influence this particular readout, 2) no other SFC metric (that they currently consider as phenomenological) can provide a readout of this model parameter even given the same circuit model, and 3) that the insight gained from the neural field model (with the restricted set of free parameters) is applicable to real data in a general way (i.e., if I applied the method to recordings from the visual cortex, would the same readout imply the same conclusion regarding circuit connectivity as for PFC? Why or why not?). And if not, these should all be discussed as limitations.

Reply: We provided a new supplementary figure, labeled Supplementary Figure 6) in the updated manuscript (for convenience we provide it in this rebuttal as Figure 4), where we vary inhibitory connectivity strength in a graded fashion and show that GPLA output scales parametrically in the following sense: the regression coefficient estimated from the phase-modulus relation is a monotonous function of the imaginary part of complex number a in Equation 9.

We now further discuss the additional requests for evidence:

- "*1) no other model parameters would strongly influence this particular readout*": this is supported by our approximate analytical expression in Equation 9, which relies on the assumptions of low frequency and synchronous exogenous input with narrow spatial width.

- "*2) no other SFC metric (that they currently consider as phenomenological) can provide a readout of this model parameter, even given the same circuit model*" We think this is hard to demonstrate and do not intend nor see a need of making such a claim due to the nature of this property. Interpretablity is beneficial only when the dependency between biophysical parameters and QoI is shown explicitly, and we argue that showing interpretability of a particular QoI is a valuable enough contribution without demonstrating that other quantities are not. We did not mean to imply that competing quantities are not interpretable, but that interpretability has not been demonstrated, as far as we know. We clarified this point in the revised manuscript, notably at the beginning of the discussion we write:

  > *In this study, we developed Generalized Phase Locking Analysis (GPLA) as — to the best of our knowledge — the first* multivariate *method with* **demonstrated biophysically interpretability** *for investigating the coupling between spatially distributed spiking and LFP activity.*

  One message of this work is to encourage scientists to look for interpretable quantities, because the more interpretable QoIs are found for the broadest range of models, the easier it will be to build mechanistic explanations.

- "3) that the insight gained from the neural field model (with the restricted set of free parameters) is applicable to real data in a general way": We believe at this point there is a consensus in

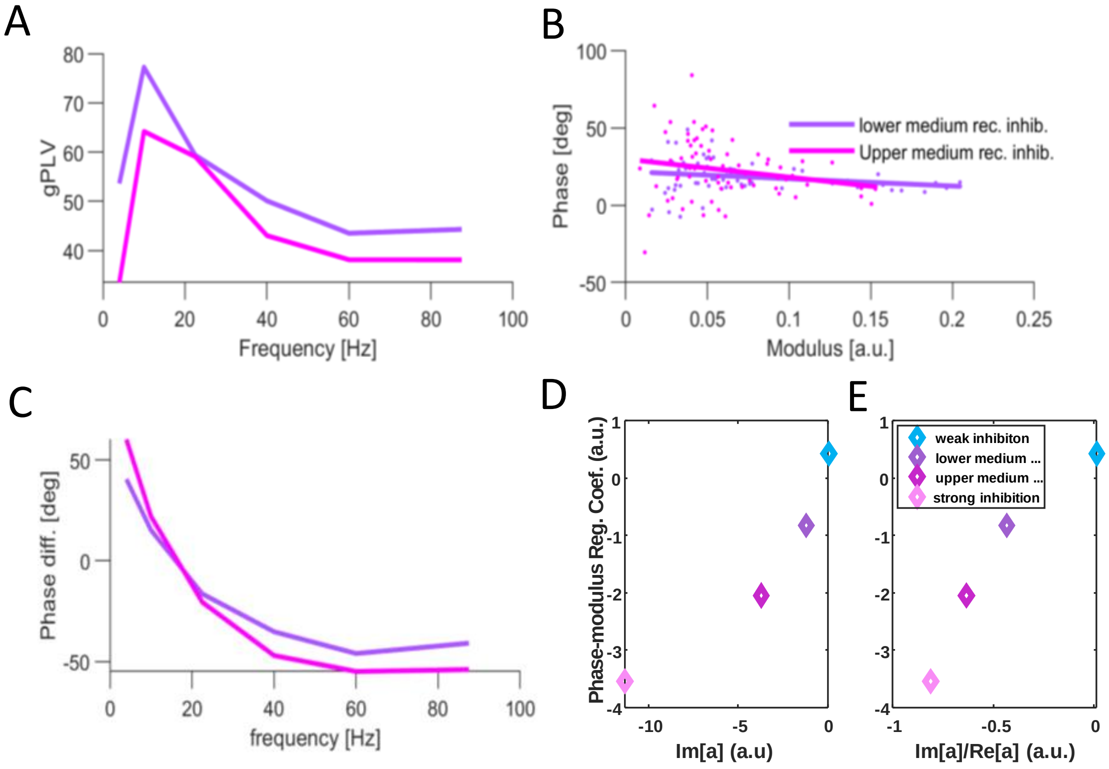

Figure 4: **Phase-modulus relation dependency on level of inhibition.**
Related to manuscript's Figure 7D. **(A)** Same as manuscript's Figure 7C. for simulations at intermediate levels of recurrent inhibition. **(B)** Same as manuscript's Figure 7D. for simulations at intermediate levels of recurrent inhibition. **(C)** Same as manuscript's Figure 7E. for simulations at intermediate levels of recurrent inhibition. **(D)** Magnitude of phase modulus regression coefficient (rescaled by $180/\pi$ to have it in radians) as a function of imaginary part of a derived from Equation 9. **(E)** Same as (A) for $\text{Im}[a]/\text{Re}[a]$ instead of $\text{Im}[a]$.

philosophy of science regarding the impossibility to provide strict guaranties that insights gained by scientific models reflect "reality", but yet those are constantly used for scientific explanation, leading to considerable work on the benefits of "idealization" for scientific explanation. As argued above, notably in point P 1.3, we have done our best to put cautionary notes and clarify the best practices we can apply in the revised manuscript.

Reviewer Point P 1.11 — *These are just a few specific examples that I believe could provide more support to the central point that gPLA provides access to mechanistic insight over existing ("phenomenological") SFC metrics, but I hope my point is clear. To reiterate, I think the framework of pairing a (novel and interesting) data analysis tool with a mechanistic model that provides different hypothesis for how the empirically observed data could've been generated is an extremely important direction for systems and cognitive neuroscience. This framework (or philosophy) of computational modeling and analysis predates this particular paper, of course, but I commend the authors for taking such an approach, and I think this paper can be reorganized to emphasize that aspect more prominently. My main contention is regarding the point that, if I'm reading it correctly, it seems that the authors claim that gPLA inherently provides mechanistic insight in ways that other SFC methods cannot, independent of the specific mechanistic model and dataset used. At the very least, the argument should be that, given a SPECIFIC mechanistic model and dataset, a method that naturally accounts for multivariate data provides a better view into the underlying circuit parameters than a univariate method (which has to be shown).*

Reply: We are very grateful to the reviewer for emphasizing the interest in our perspective and highlighting its potential contribution to the community. We believe the reviewer's concerns have been addressed in the above replies.

Reviewer Point P 1.12 — *A more general note about the presentation: currently, the paper reads a bit like a collection of nice but somewhat unrelated projects that use the same analysis method on vastly different computational models. It's hard to say if there is a central point that these analyses all go towards supporting, or if there is a connection between the different model choices. If the intent is simply to demonstrate how gPLA may work with models of varying complexity to extract biological insight, then I would recommend explicitly stating so in the abstract or early in the introduction, and emphasize that the broad applicability of gPLA is a feature (but why that is not true of other SFC methods, even univariate ones). Perhaps it's that gPLA depends on certain assumptions of spatial structure about the model / data-generating circuit, which is what allows it to extract that information? On the other hand, if there is a high level point that all the models support, I would recommend making that and the connection between them explicit (i.e., why they were chosen). I'm sorry that I don't have a more concrete recommendation on the writing, but my feedback as a reader is that it was quite overwhelming to go through the manuscript even though I am familiar with most of the analysis and modeling techniques.*

Reply: Thank you for providing hints on unclear aspects. Our purpose for using a variety of models was to demonstrate different aspects of GPLA with appropriate models. For instance, we used toy models to illustrate the idea of the method in the simplest possible way; and use a simple generative model for spike and LFP (rather than a mean-field model), to demonstrate the statistical benefit of GPLA. Thus, we chose the models with the appropriate levels of complexity required for the message the analysis

intends to convey. This is briefly mentioned in this abstract and, to reflect the logic, we have revised an introductory paragraph as follows:

> *" The benefits of this approach are demonstrated in detail with network models with increasing levels of complexity and biophysical realism, and ultimately with neural data. Each of these settings is designed to demonstrates certain strengths of GPLA. First we provide a theoretical motivation and illustrate how to interpret the outcome of the analysis with toy models. Then we illustrate the statistical benefits of GPLA over uni-variate methods with several simple generative models of spike and LFP. Thereafter, we turn to biophysical interpretability of GPLA using an analytical reduction of two population (excitatory-inhibitory) neural field models. This mechanistic interpretation is exemplified in computational models of hippocampal and cortical neural networks. In particular, we show how studying the phase of GPLA can untangle the contribution of recurrent interactions to the observed spatio-temporal dynamics. Based on these results, application of GPLA to Utah array recordings finally provides evidence of strong feedback inhibition in the macaque prefrontal cortex."*

Most importantly, we have now introduced an explicit definition of biophyisical interpretability at the begining of the result section (together with the illustrative Figure 1A), which we use as a guiding principle along the manuscript.

> *" The overarching motivation of this work is to foster a neuroscientific understanding of experimental data by leveraging biophysical models, i.e. models that comprise equations accounting for the biophysics of neural activity and measurements. Broadly construed, models can be ranked according to the chosen trade-off between realism and complexity, with on one end simplified (e.g. low dimensional, linear, ...) analytically tractable models, whose dependency on biophysical properties is easily characterized, and on the opposite end, highly detailed computational models, where the role of biophysical parameters can only be assessed by running costly simulations. However, when it comes to the use of models for interpreting data, another key aspect coming into play is the choice of quantities of interest (QoI) (following Transtrum and Qiu [2016]), which are used to assess qualitatively or quantitatively the match between experimental data and candidate models. Indeed, some QoIs may be easier to interpret with a given biophysical model than others, in the sense that they have a more straightforward dependency on biophysical parameters. This leads us to introducing the notion of biophysical interpretability, illustrated in Figure ??A and defined as follows: a QoI is biophysically interpretable for a particular model whenever we can identify biophysical properties of the model that influence the QoI in a simple way (the simpler, the more interpretable). By "simple", we refer to the low complexity of the functional relationship between the properties and the QoI. To fix ideas, functional relations containing only few biophysical parameters and monotonous functions will be considered simple, although the choice of notion of complexity may be adapted to the case at hand. This interpretability property clearly depends on the model employed, and its benefits for analyzing experimental data relies on the assumption that the chosen model captures well relevant properties of the ground truth mechanism, as it is the case for any attempt at understanding empirical data through mechanistic models. The validity of this assumption can never be fully guarantied and which aspects to incorporate in the model to address a particular question should be assessed based on the literature. In this work, we rely on neural field models [Jirsa and Haken, 1996], which lend themselves to analytical treatment,*

while still accounting for key aspects of the underlying biophysical network mechanisms. As neurophysiology experiments rely on an increasing number of recording channels, the choice of QoI has to be made from a space of increasingly large dimensions, and we argue that the notion of biophysical interpretability can guide this choice. Specifically, we will consider QoIs that quantify SFC, and a multidimensional generalization of it."

References

G. Ashida, H. Wagner, and C. E. Carr. Processing of Phase-Locked Spikes and Periodic Signals. In *Analysis of Parallel Spike Trains*, Springer Series in Computational Neuroscience, pages 59–74. Springer, Boston, MA, 2010. ISBN 978-1-4419-5674-3 978-1-4419-5675-0. doi: 10.1007/978-1-4419-5675-0_4.

C. Börgers and N. Kopell. Synchronization in networks of excitatory and inhibitory neurons with sparse, random connectivity. *Neural computation*, 15(3):509–538, 2003.

G. Buzsáki. Large-scale recording of neuronal ensembles. *Nature Neuroscience*, 7(5):446–451, May 2004. ISSN 1546-1726. doi: 10.1038/nn1233.

C. Craver. Constitutive explanatory relevance. *Journal of Philosophical Research*, 32:3–20, 2007.

A. S. Dickey, A. Suminski, Y. Amit, and N. G. Hatsopoulos. Single-Unit Stability Using Chronically Implanted Multielectrode Arrays. *Journal of Neurophysiology*, 102(2):1331–1339, Aug. 2009. ISSN 0022-3077. doi: 10.1152/jn.90920.2008.

B. Ermentrout and D. Pinto. Neurophysiology and waves, 2007. URL `http://www.siam.org/news/news.php?id=1091`.

G. B. Ermentrout and D. Kleinfeld. Traveling electrical waves in cortex: insights from phase dynamics and speculation on a computational role. *Neuron*, 29(1):33–44, 2001.

M. Fukushima, Z. C. Chao, and N. Fujii. Studying brain functions with mesoscopic measurements: Advances in electrocorticography for non-human primates. *Current Opinion in Neurobiology*, 32: 124–131, June 2015. ISSN 0959-4388. doi: 10.1016/j.conb.2015.03.015.

P. Gao and S. Ganguli. On simplicity and complexity in the brave new world of large-scale neuroscience. *Current Opinion in Neurobiology*, 32:148–155, June 2015. ISSN 0959-4388. doi: 10.1016/j.conb.2015.04.003.

S. Grün. Data-Driven Significance Estimation for Precise Spike Correlation. *Journal of Neurophysiology*, 101(3):1126–1140, Mar. 2009. ISSN 0022-3077. doi: 10.1152/jn.00093.2008.

H. Jiang, A. Bahramisharif, M. A. J. van Gerven, and O. Jensen. Measuring directionality between neuronal oscillations of different frequencies. *NeuroImage*, 118:359–367, Sept. 2015. ISSN 1053-8119. doi: 10.1016/j.neuroimage.2015.05.044.

V. K. Jirsa and H. Haken. Field Theory of Electromagnetic Brain Activity. *Phys. Rev. Lett.*, 77 (5):960–963, July 1996. doi: 10.1103/PhysRevLett.77.960.

A. L. Juavinett, G. Bekheet, and A. K. Churchland. Chronically implanted Neuropixels probes enable high-yield recordings in freely moving mice. *eLife*, 8:e47188, Aug. 2019. ISSN 2050-084X. doi: 10.7554/eLife.47188.

J. J. Jun, N. A. Steinmetz, J. H. Siegle, D. J. Denman, M. Bauza, B. Barbarits, A. K. Lee, C. A. Anastassiou, A. Andrei, C. Aydin, M. Barbic, T. J. Blanche, V. Bonin, J. Couto, B. Dutta, S. L. Gratiy, D. A. Gutnisky, M. Hausser, B. Karsh, P. Ledochowitsch, C. M. Lopez, C. Mitelut, S. Musa, M. Okun, M. Pachitariu, J. Putzeys, P. D. Rich, C. Rossant, W. L. Sun, K. Svoboda, M. Carandini, K. D. Harris, C. Koch, J. O'Keefe, and T. D. Harris. Fully integrated silicon probes for high-density recording of neural activity. *Nature*, 551:232–236, Nov. 2017. ISSN 1476-4687 (Electronic) 0028-0836 (Linking). doi: 10.1038/nature24636.

I. Lakatos. Falsification and the methodology of scientific research programmes. In *Can theories be refuted?*, pages 205–259. Springer, 1976.

Z. Li, D. Cui, and X. Li. Unbiased and robust quantification of synchronization between spikes and local field potential. *J Neurosci Methods*, 269:33–8, Aug. 2016. ISSN 1872-678X (Electronic) 0165-0270 (Linking). doi: 10.1016/j.jneumeth.2016.05.004.

T. O'Leary, A. C. Sutton, and E. Marder. Computational models in the age of large datasets. *Current Opinion in Neurobiology*, 32:87–94, June 2015. ISSN 0959-4388. doi: 10.1016/j.conb.2015.01.006.

B. Pesaran, M. Vinck, G. T. Einevoll, A. Sirota, P. Fries, M. Siegel, W. Truccolo, C. E. Schroeder, and R. Srinivasan. Investigating large-scale brain dynamics using field potential recordings: Analysis and interpretation. *Nature Neuroscience*, page 1, June 2018. ISSN 1546-1726. doi: 10.1038/s41593-018-0171-8.

J. F. Ramirez-Villegas, K. F. Willeke, N. K. Logothetis, and M. Besserve. Dissecting the synapse- and frequency-dependent network mechanisms of in vivo hippocampal sharp wave-ripples. *Neuron*, 100(5):1224–1240.e13, Dec 2018. doi: 10.1016/j.neuron.2018.09.041.

W. C. Salmon. *Scientific explanation and the causal structure of the world.* Princeton University Press, 1984.

I. H. Stevenson and K. P. Kording. How advances in neural recording affect data analysis. *Nature Neuroscience*, 14(2):139–142, Feb. 2011. ISSN 1546-1726. doi: 10.1038/nn.2731.

M. K. Transtrum and P. Qiu. Bridging mechanistic and phenomenological models of complex biological systems. *PLoS computational biology*, 12(5):e1004915, 2016.

R. D. Traub, M. A. Whittington, E. H. Buhl, J. G. Jefferys, and H. J. Faulkner. On the mechanism of the gamma –¿ beta frequency shift in neuronal oscillations induced in rat hippocampal slices by tetanic stimulation. *The Journal of Neuroscience*, 19(3):1088–1105, Feb 1999.

M. Vinck, M. van Wingerden, T. Womelsdorf, P. Fries, and C. M. Pennartz. The pairwise phase consistency: A bias-free measure of rhythmic neuronal synchronization. *Neuroimage*, 51(1):112–22, May 2010. ISSN 1095-9572 (Electronic) 1053-8119 (Linking). doi: 10.1016/j.neuroimage.2010.01.073.

M. Vinck, F. P. Battaglia, T. Womelsdorf, and C. Pennartz. Improved measures of phase-coupling between spikes and the Local Field Potential. *J Comput Neurosci*, 33(1):53–75, Aug. 2012. ISSN 1573-6873 (Electronic) 0929-5313 (Linking). doi: 10.1007/s10827-011-0374-4.

M. A. Whittington, R. D. Traub, N. Kopell, B. Ermentrout, and E. H. Buhl. Inhibition-based rhythms: experimental and mathematical observations on network dynamics. *International journal of psychophysiology*, 38(3):315–336, 2000.

R. C. Williamson, B. Doiron, M. A. Smith, and B. M. Yu. Bridging large-scale neuronal recordings and large-scale network models using dimensionality reduction. *Current Opinion in Neurobiology*, 55:40–47, Apr. 2019. ISSN 0959-4388. doi: 10.1016/j.conb.2018.12.009.

M. Zarei, M. Jahed, and M. R. Daliri. Introducing a comprehensive framework to measure spike-lfp coupling. *Frontiers in Computational Neuroscience*, 12, 2018. ISSN 1662-5188. doi: 10.3389/fncom.2018.00078.

M. Zeitler, P. Fries, and S. Gielen. Assessing neuronal coherence with single-unit, multi-unit, and local field potentials. *Neural Comput*, 18:2256–81, Sept. 2006. ISSN 0899-7667 (Print) 0899-7667 (Linking). doi: 10.1162/neco.2006.18.9.2256.
